# The CRE1 carbon catabolite repressor of the fungus *Trichoderma reesei: *a master regulator of carbon assimilation

**DOI:** 10.1186/1471-2164-12-269

**Published:** 2011-05-27

**Authors:** Thomas Portnoy, Antoine Margeot, Rita Linke, Lea Atanasova, Erzsébet Fekete, Erzsébet Sándor, Lukas Hartl, Levente Karaffa, Irina S Druzhinina, Bernhard Seiboth, Stéphane Le Crom, Christian P Kubicek

**Affiliations:** 1 IFP Energies nouvelles, Département Biotechnologie, 1-4 Avenue de Bois-Préau, 92852 Rueil-Malmaison Cedex, France; 2 École normale supérieure, Institut de Biologie de l'ENS, IBENS, Paris, F-75005 France. Inserm, U1024, Paris, F-75005 France. CNRS, UMR 8197, Paris, F-75005 France; 3 Austrian Center of Industrial Biotechnology, c/o Institute of Chemical Engineering, Technische Universität Wien, Getreidemarkt 9/166, A-1060 Vienna, Austria; 4 Research Area Gene Technology and Applied Biochemistry, Institute of ChemicalEngineering, Technische Universität Wien, Getreidemarkt 9/166, A-1060 Vienna, Austria; 5 Department of Biochemical Engineering, Faculty of Science and Technology, University of Debrecen, H-4010, P.O.Box 56, Debrecen, Hungary; 6 Department of Plant Protection, Faculty of Agriculture and Food Sciences and Environmental Management, University of Debrecen, H-4032 Böszörményi út 138., Debrecen, Hungary

## Abstract

**Background:**

The identification and characterization of the transcriptional regulatory networks governing the physiology and adaptation of microbial cells is a key step in understanding their behaviour. One such wide-domain regulatory circuit, essential to all cells, is carbon catabolite repression (CCR): it allows the cell to prefer some carbon sources, whose assimilation is of high nutritional value, over less profitable ones. In lower multicellular fungi, the C2H2 zinc finger CreA/CRE1 protein has been shown to act as the transcriptional repressor in this process. However, the complete list of its gene targets is not known.

**Results:**

Here, we deciphered the CRE1 regulatory range in the model cellulose and hemicellulose-degrading fungus *Trichoderma reesei *(anamorph of *Hypocrea jecorina*) by profiling transcription in a wild-type and a delta-*cre1 *mutant strain on glucose at constant growth rates known to repress and de-repress CCR-affected genes. Analysis of genome-wide microarrays reveals 2.8% of transcripts whose expression was regulated in at least one of the four experimental conditions: 47.3% of which were repressed by CRE1, whereas 29.0% were actually induced by CRE1, and 17.2% only affected by the growth rate but CRE1 independent. Among CRE1 repressed transcripts, genes encoding unknown proteins and transport proteins were overrepresented. In addition, we found CRE1-repression of nitrogenous substances uptake, components of chromatin remodeling and the transcriptional mediator complex, as well as developmental processes.

**Conclusions:**

Our study provides the first global insight into the molecular physiological response of a multicellular fungus to carbon catabolite regulation and identifies several not yet known targets in a growth-controlled environment.

## Background

Many filamentous fungi have developed a predominantly saprobic lifestyle, in which successful competition with other microorganisms for the limited resources present in the environment is the key for survival. To this end mechanisms evolved that allow a rapid adaption to changing nutrient conditions. One such wide-domain regulatory circuit is carbon catabolite repression (CCR): it allows the preferred assimilation of carbon sources of high nutritional value over others [1-4]. This is usually achieved through inhibition of gene expression of enzymes involved in the catabolism of other carbon sources than the preferred ones. In multicellular ascomycetes, the C2H2 type transcription factor CreA/CRE1*, which is related to Mig1/Mig2/Mig3 proteins that mediate glucose repression in *Saccharomyces cerevisiae *[5] and to the mammalian Krox20/Egr and Wilm's tumour proteins [6], has been shown to act as a repressor mediating CCR [7,8]. CreA/CRE1 binds to the promoters of the respective target genes via the consensus motif 5'-SYGGRG-3', whose function *in vivo *has been shown both in *Aspergillus nidulans *and *Trichoderma reesei *[9-12]. Functional CreA/CRE1 binding sites frequently consist of two closely spaced 5'-SYGGRG-3'motifs, and it has been suggested that direct repression would only occur through such double binding sites [10,11]. In addition, phosphorylation of a serine in a conserved short stretch within an acidic domain of *T. reesei *CRE1 has been demonstrated to regulate its DNA binding [13].

Today, a plethora of genes have been shown to be under control of CreA or CRE1 (reviewed in [7]) but the mechanisms triggering regulation by CreA/CRE1 are less well understood. In *A. nidulans*, regulation by CreA can be initiated by several so called "repressing" hexoses, requires their phosphorylation, and is affected by the growth rate [14-16]. Most studies on CCR in fungi have been made with gene model systems where CCR functions in the counteraction of gene induction [7]. In contrast, little information is available about which genes directly respond to a relief from CCR. Since *creA/cre1*-knock out mutants display severe phenotypic changes such as reduced growth, abnormal hyphal morphology and sporulation [17,18], such studies are only possible under carefully controlled conditions. Here we chose to use chemostat cultures on D-glucose as a carbon source at two different growth rates (one repressing and one derepressing [16]) to investigate the genome-wide changes in gene expression in relation to CRE1 function, using a *Δcre1 *recombinant mutant strain of *T. reesei *and corresponding control strain.

* Footnote: we accept the gene/protein nomenclature of Sordariomycetes and therefore name the *Trichoderma *CreA orthologue CRE1.

## Results

### Construction and phenotypic characteristics of a *cre1 *knock-out strain of *T. reesei*

We constructed a *Δcre1 *recombinant strain of *T. reesei *QM 9414 by replacing its ORF and part of its 5'-and 3'-nt regions by a hygromycin B resistance gene [19] under constitutive expression signals. In accordance with Nakari-Setälä *et al. *[18], the corresponding knock-out strains exhibited a reduced radial growth rate on plates, and formed smaller colonies, fewer aerial hyphae and less spores. In addition, the *Δcre1 *strain displayed shorter but more robust hyphae, that contained a considerably thickened cell wall and less septa (Additional File 1: Figure S1 and Table S1). All these mutant phenotypes were eliminated by retransforming the *Δcre1 *strain with the *cre1 *gene, thus proving that they are *cre1 *specific (data not shown). We used phenotype microarrays to analyze whether the above noted reduction in the growth rate is general or specific for some carbon sources only. The results showed that - in contrast to the radial growth on plates - the Δ*cre1 *strain grew significantly slower on only 5 of 95 carbon sources (Glycogen -37 [±4], p = 0.004; arbutin -27 [±4], p = 0.0006; adenosine -42 [±5], p = 0.022; salicin -34 [±4], p = 0.007; and amygdalin -42 [±6], p = 0.013), but on the other hand was unaffected on the majority of them (within ± 25% of variation, Chi square test p > 0.05; see Additional File 1, Figure S2). However, increased growth of > 30% of the control, which would be expected if CRE1 represses growth on a given carbon source, was observed for 9 carbon sources shown in Additional File 1, Figure S3 (D-galactose +38 [±3], p = 0.0008; L-sorbose +34 [±3], p = 0.027; D-xylose +48 [±5], p = 0.002; palatinose +31 [±6], p = 0.033; maltose +58 [±6], p = 0.002; stachyose +45 [±4], p = 0.03; xylitol +37 [±4], p = 0.017; adonitol +46 [±6], p = 0.0008); and glucuronamide +73 [±3], p = 0.044).

### Wide domain regulation by CRE1: experimental design and properties

**Figure 1:**
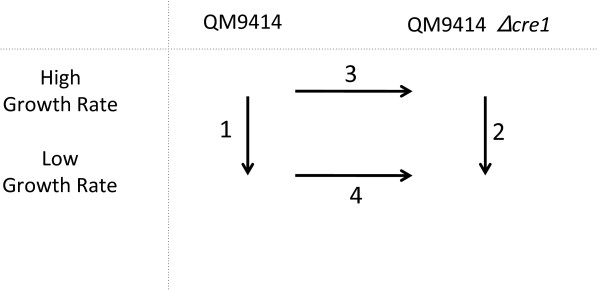
**Experimental design used to study the role of CRE1 and growth rate on gene expression in *T. reesei***. Two strains were compared, i.e. *T. reesei *QM 9414 (as the reference strain) and a *Δcre1 *strain derived from it. In addition, two growth rates (which have previously been shown to lead to CCR repression and derepression, respectively) were compared: D = 0.07 h^-1 ^, and D = 0.025 h^-1 ^. Two dye switch hybridizations were performed. The position of the arrow points to the experimental condition that was used as "result" (i.e. in condition 1, the changes in expression levels at the low growth rate when compared with those at the high growth rate are given). The numbers over the arrows refer to the experiment numbers used further in text, figures and tables.

**Figure 2:**
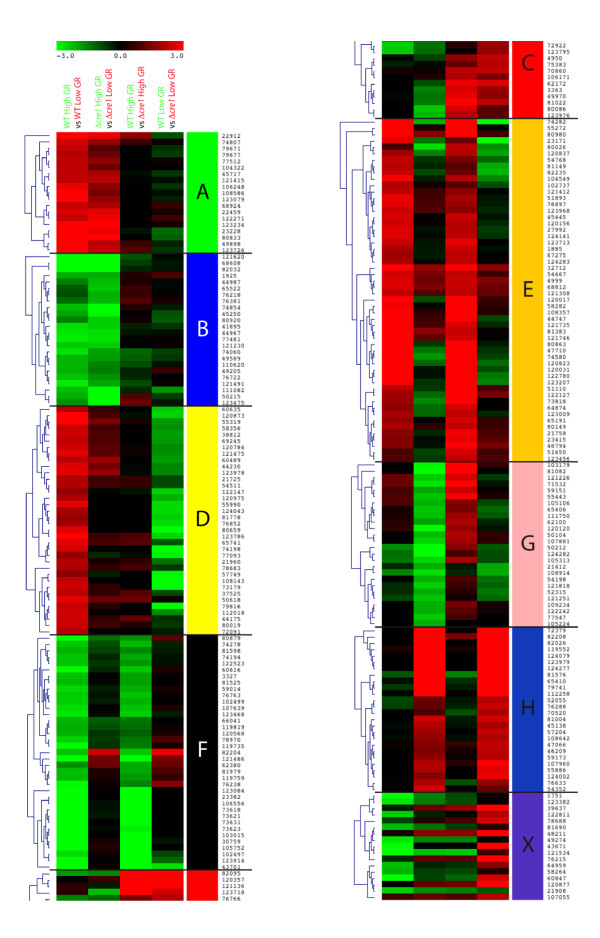
**Heat map of strongly expressed genes**. Heat map displaying the result from hierarchical clustering of the strongly regulated genes. The colored vertical bars give the letter identifying the respective clusters. Genes contained in the clusters are given by the ID of the encoded proteins. GR: growth rate; wt: QM 9414 wild-type strain.

**Figure 3:**
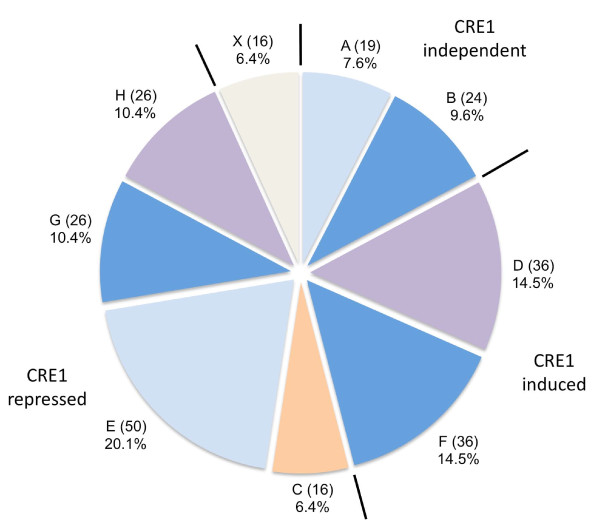
**Distribution of genes among expression clusters**. Nine clusters, grouped according to their regulation by CRE1 (i.e. repressed, induced, or independent) were obtained from the microarray results (from A to H, and X). The color code indicates the effect of the growth rate: blue specifies clusters of genes affected only by a high growth rate (dark blue indicates upregulation, light blue downregulation). Purple specifies the genes upregulated at a low growth rate. Gene clusters whose expression is not influenced by the growth rate are given in orange. The percentages indicate the fraction of the total genes that is present in the respective cluster and the number of genes in each cluster is specified in parentheses.

We have used whole-genome DNA microarrays with the goal of identifying the main genes in *T. reesei *that are controlled by CRE1. Since carbon catabolite repression is known to be dependent on the growth rate, the microarray experiments were therefore performed in chemostat cultures at two constant growth rates that were earlier shown [16] to be carbon repressing (0.07 h^-1^) and potentially carbon derepressing (0.025 h^-1^), respectively (Figure 1). After data pretreatment and normalization, we applied the linear modeling approach and the Bayes statistics implemented in the limma R package [20] to our biological replicates as described in the Methods part. Using these criteria we retrieved a list of 251 genes whose expression was regulated in at least one of the four experimental conditions. One of them was *cre1 *itself (in the parent strain), which is trivial and was omitted from all further investigations. The (in part overlapping) occurrence of the truly regulated 250 genes under the four different experimental conditions is shown in Additional File 1, Figure S4. We used clustering algorithms (see Methods) to divide them into 9 different clusters (Figure 2). The effect of CRE1 and its interplay with the growth rate is summarized in Figure 3: 47.3% of the identified genes are in fact repressed by CRE1, but 29.0% are CRE1 induced and 17.2% are CRE1 independent. In addition, 62.2% of the genes are influenced by the high growth rate (34.5% induced and 27.7% repressed), 24.9% are derepressed at the low growth rate and 6.4% are growth rate independent. A complete list of all genes is given in Additional File 1, Table S2. To confirm these microarray results, quantitative Real-Time-PCR (qRT-PCR) was performed on a subset of the genes belonging to different clusters. The gene for isocitrate lyase was included in this set, as its regulation by CRE1-mediated carbon catabolite repression is known ([16], and references therein) and its appearance in our dataset confirms that the growth rates were chosen appropriately. As shown in Additional File 1, Table S3, these genes that showed differential expression between two or more of the used conditions in the microarray study were also differentially expressed in the same direction upon Real Time-PCR analysis. We therefore conclude that the microarray expression ratios indeed reflect differences in the expression of these genes.

Up and down regulated genes were annotated and categorized as described in the Methods section. The enrichment for the major FunCat categories was assessed in each cluster, and a Gene Ontology (GO) enrichment analysis was also done on the whole dataset as further control (see Additional File 1, Tables S4 and S5).

### Genes upregulated in the absence of CRE1 function in *T. reesei*

As shown in Figure 3, genes which were upregulated in the Δ*cre1 *mutant strain could be grouped into four classes: genes that were upregulated in the CRE1 knockout independently of the growth rate (cluster C, 16 genes); genes upregulated in the CRE1 knockout at high growth rate only (cluster E, 50 genes); genes for which CRE1 function counteracted an induction at high growth rate (cluster G, 26 genes); and genes for which CRE1 function counteracted an induction at low growth rate (cluster H, 26 genes).

**Figure 4:**
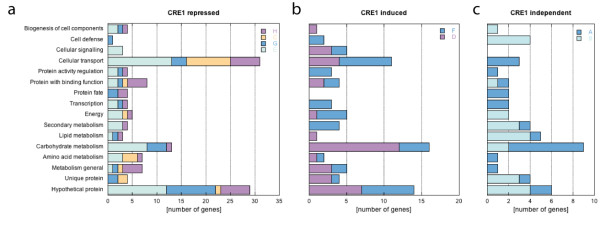
**Distribution of genes among the main functional categories (FunCat)**. The number of genes belonging to categories is presented for each cluster, with the same color code as in Figure 3. Subfigures distinguish the genes following the same classification as in the text: genes upregulated in the absence of CRE1 function (a), genes downregulated in the absence of CRE1 function (b), and genes regulated only by the growth rate but not by CRE1 (c).

Figure 4a shows the distribution of functional categories (FunCat) within these 4 gene clusters. Genes which have orthologues in other fungi but for which no function can be predicted were most abundant in clusters E and G. Genes for carbohydrate degradation were most abundant in the cluster that groups together the genes upregulated in the CRE1 knockout at high growth rate (cluster E). In this cluster, we detected a significant enrichment of genes involved in cellular transport (see Table S4). This category was also significantly enriched in cluster C, which comprises the genes upregulated in the CRE1 knockout independently of the growth rate. The nature of cellular transport proteins was diverse in cluster E, but was dominated by permeases for transport of nitrogenous compounds in cluster C.

The cluster of the genes upregulated in the CRE1 knockout at high growth rate (E) also exhibited genes linked to transcription regulation (e.g. the helicase SNF2 involved in chromatin remodeling and the transcriptional regulator MedA involved in sporulation), as well as PhiA and RAS1, both participating in hyphal development.

This latter function was also represented with the *bys1 *transcript in the cluster of genes for which CRE1 function counteracted an induction at high growth rate (cluster G). This cluster comprised different genes linked to carbohydrate metabolism (e.g. glycoside hydrolases and glycoside transferases), as well as *hsp30*, a single integral plasma membrane heat shock protein that is induced by several stresses, including glucose limitation [21].

As found for cluster E, the cluster that groups together genes for which CRE1 function counteracted an induction at low growth rate (cluster H) exhibited a majority of genes involved in cellular transport. A membrane protein linked to sporulation, CMP1, has also been found in this cluster. The significance of the enrichment of these functional categories has been verified using Gene Ontology annotation (Additional File 1, Table S5).

Genes contained in cluster X exhibited a heterogenous pattern of gene expression, but their expression was consistently highest in the Δ*cre1 *strain at low growth rates, and lowest in the parental strain at high growth rates. We therefore conclude that genes of this cluster are also subject to CRE1-dependent CCR. Gene with putative functions did not reveal any common characteristics. However, we detected the orthologue of *A. nidulans creD *(*cre4)*, which encodes a protein with arrestin and PY motifs known to interact with ubiquitin ligase [22]. Disruption of *creD *in *A. nidulans *confers partial relief from CCR [23].

### Genes downregulated in the absence of CRE1 function in *T. reesei*

The expression of a smaller number of genes was decreased in the Δ*cre1 *strain, either only at the low (cluster D, 36 genes) or the high growth rate (cluster F, 36 genes), suggesting that CRE1 acts - either directly or indirectly - positively on these genes. The highest number of genes from the cluster that groups together the genes downregulated in the CRE1 knockout at high growth rate (F) corresponds to genes that encode proteins involved in cellular transport or proteins for which no function has been characterized (Figure 4b). This cluster also comprised 4 genes for carbohydrate metabolism, 2 synthases and 2 oxidoreductases involved in secondary metabolite formation, as well as 3 transcription factors with Zn2Cys6 and bZIP DNA binding domains. Cluster D, which comprise genes downregulated in the CRE1 knockout at low growth rate, exhibited a significant enrichment of genes encoding carbohydrate active enzymes. This cluster also exhibited a broad composition of other various gene categories, those encoding unknown proteins and compounds involved in cellular transport being most abundant.

### Genes regulated only by the growth rate but not by CRE1

17.2% of the genes detected in this study displayed an expression pattern that was independent of CRE1 function, i.e. their expression was different only between the two growth rates, but not in any direct comparison between the parent and the *Δcre1 *mutant strain. 24 of them were activated (cluster B), whereas 19 (cluster A) were actually repressed by a high growth rate. A significant enrichment of genes for carbohydrate metabolism has been detected in the cluster of genes repressed by the high growth rate (cluster A), whereas the group of genes induced at high growth rate (cluster B) comprised the whole set of genes needed to catabolize N-acetylglucosamine to fructose-6-phosphate and exhibited five genes dedicated to fatty acid metabolism (Figure 4c; see also Additional File 1, Tables S4 and S5 for enrichment statistics).

### Presence of CRE1-binding sites in the 5'-region of the identified genes

In order to link the diverse regulatory effects of CRE1 identified in this study to a direct gene target, we looked for the CRE1 binding pattern 5'-SYGGRG-3' [10,11] in sequences 1 kb upstream of the coding region of the genes that were up-or down-regulated in the microarray experiments. For each cluster, an average number of sites per gene was calculated and normalized to the average number of sites per gene found in the whole *T. reesei *genome. Since binding of CreA/CRE1 has been described to require two adjacent binding sites, or may be clustered in *cis*-regulatory element enriched regions (CRERs, [24]), we also looked for the occurrence of direct or inverted repeats (motif pairs). The total number of these "paired motifs" was calculated for each gene, and an average per gene was again measured for each cluster, and then normalized to the average number in the genome. Two spacing distances between the two motifs were tested, which were maximally 20 bp or 50 bp. For a value of 50 bp, the average number of complex motifs per gene for the whole genome was 0.9 while it was 0.5 for a 20 bp distance value. For each motif pair alone, values ranged from 0.1 to 0.5. Significance of the differences in average per cluster compared to the average on the whole genome was assayed with bi-directional Student t-test. The relatively high frequency of the motifs, combined with the possible presence of genuine primary and secondary CreA/CRE1 target, resulted in often high standard deviation, making statistical validation difficult. However, the results (Table 1) show that the CRE1 binding motif, was significantly more abundant in the CRE1 repressed cluster E, and in the CRE1 activated cluster D. This observation was less clear for cluster H (genes for which CRE1 function counteracted an induction at low growth rate), while no enrichment was detected for cluster F and G (genes downregulated in the CRE1 knockout at high growth rate, and genes for which CRE1 function counteracted an induction at high growth rate, respectively). While we were not able to statistically validate this, a trend in enrichment is visible for cluster C, which groups together genes that were upregulated in the CRE1 knockout independently of the growth rate. These results suggest that clusters D, E and maybe C are enriched in direct CRE1 targets while clusters F and H are indirect results of the CRE1 regulation. In support of this conclusion, the values obtained for the CRE1 independent genes contained in clusters A and B were in the range of (or even below) the genomic average.

**Table 1:** Results from the promoter analysis

		SYGGRG ... CYCCRS			
		CYCCRS ... SYGGRG			
		SYGGRG ... SYGGRG			
		SYGGRG			SAGGGRGR
		20 bp distance		50 bp distance	
Average number per gene in whole genome		4.13	0.47	0.9	0.57
	A	-8%	- 66%**	-24%	-17%
	B	- 3%	- 2%	- 7%	17%
	C	26%	33%	11%	34%
Enrichment in clusters	D	+23%**	36%	+48%**	+105%**
	E	+16%**	+58%*	26%	+72%**
	F	- 3%	9%	11%	-15%
	G	1%	- 43%*	- 6%	-26%
	H	1%	+23%*	3%	8%
	X	-16%	- 50%**	-28%	24%

Enrichments compared to whole genome values are shown.

Results of statistical tests (Student t-test) are indicated as follow:

* p-value between 0.05 and 0.1;

** p-value < 0.05. Values in bold are significant positive enrichments, which are predominantly discussed in the text.

We also looked for the presence of nucleotide motifs that are shared by genes regulated by CRE1, as described in the Methods section. One of the detected motif using the RSA-Tools software (5'-SAGGGRG-3') was indeed significantly enriched in clusters D and E (i.e. the clusters most enriched in CRE1 motifs). This motif is otherwise found at least once at a 57% frequency in all *T. reesei *promoters.

## Discussion

The carbon catabolite regulator CreA/CRE1 is one of a few wide-domain master regulators identified in multicellular fungi, and has been shown to govern the repression of genes involved in polysaccharide degradation and in the utilization of ethanol and amino acids as carbon sources [7,8]. However, the major drawback inherent to these studies is that the growth rate was not controlled. Using chemostat cultures, we show here that - depending of the gene considered - the function of *T. reesei *CRE1 can be dependent on the growth rate, thus implying that the physiology of the fungal cell does affect the function of CRE1 in different ways for different genes. In addition, we show that in 29.0% of the detected genes, CRE1 actually activates (rather than represses) their expression. This number is similar to CreA-activated genes found in *A. nidulans*, although the latter investigated only a subset of the genome [25]. Our data therefore illustrate that the function of CRE1 is more complex than previously thought and clearly goes beyond CCR alone. CRE1 should therefore rather be considered a master regulator of carbon metabolism that adjusts gene regulation in relation to the rate of glucose assimilation.

Since the genome of *T. reesei *contains 9,129 predicted genes [26], the 250 highly differentially regulated genes we detected account for 2.8%, which may be considered only a low percentage. However, this study did not detect those genes where CRE1 interferes with the induction but not with basal transcription (e.g. cellulases and hemicellulases, ethanol catabolism [7], see below); the number of genes actually controlled by CRE1 is therefore probably larger and in many cases carbon-source-dependent. In this context, it is interesting to note that an orthologue of Med2, a component of the mediator complex that is conserved in all eukaryotes [27], was found to be repressed by CRE1. This protein complex mediates signals between enhancer-bound factors (activators) and the core transcriptional machinery. In *S. cerevisiae*, Med2 - together with Gal11 - has been shown to mediate the strongest activations [28], thus making them being logical targets for regulation. The identification of *T. reesei med2 *as a CCR-repressed gene may hence be interpreted as an effective means by which the cell controls expression of a broad set of genes when a drop-down in the growth rate renders their induced expression uneconomic.

207 of the genes identified in this study were in fact regulated by CRE1, whereas the other 43 only influenced by the growth rate independently of CRE1 function. 118 of the CRE1-regulated genes appeared to be repressed by CRE1. Besides unknown proteins, genes encoding membrane permeases represent the highest portion indicating that *T. reesei *carbon catabolite repression acts preferentially at the entry of substrates into the cell. Carbon catabolite repression of a high-affinity hexose permease is known in *A. nidulans *[29], and constitutes a mechanism by which fungi can retrieve even traces of high value carbon sources at a high rate. Interestingly, most of the permeases transporting nitrogenous compounds were regulated by CRE1 in a growth-rate independent manner, suggesting that proteins and their degradation products are among the preferred substitutes for fast metabolizable carbohydrates. A similar increase in amino acid uptake upon CCR has been reported for *S. cerevisiae *[30], but the effect was MIG1 independent in this case. Since the natural habitat of *T. reesei *(decaying wood) is poor in nitrogen but also in repressing carbon sources, this mechanism may enable the fungus to recruit available nitrogenous compounds at an enhanced rate.

There is a general believe that the transcription of genes encoding extracellular hydrolases (CAZome) are repressed by CRE1. Although this was in fact shown for several such genes in this study, they constituted only a very minor portion of the *T. reesei *CAZome (200 genes). Particularly, although the regulation of e.g. cellulolytic and xylanolytic enzymes by CRE1 has been demonstrated earlier [10,18], none of these genes was strongly upregulated in the Δ*cre1 *mutant. These data are in agreement with the findings that CRE1 mediated carbon catabolite repression mainly affects their induction but not their basal expression [31].

We and others have previously shown that loss-of-function of CRE1 or CreA leads to an alteration in nucleosome repositioning upon addition of glucose [32-34]. Our detection of *snf2 *as a gene subject to repression by CRE1 at a high growth rate offers an explanation for this finding: Snf2 is a component of the yeast Swi/Snf multisubunit chromatin remodelling complex [35], one of the cellular mechanisms altering chromatin structure by modulating DNA-histone interactions [36]. Swi/Snf is required for the transcriptional regulation of about 5% of the total yeast genome [37] and Snf2 plays an essential role in it by associating with nucleosomes two helical turns from the dyad axis [38].

It has also previously been reported that a loss-of-function mutation in *creA*/*cre1 *leads to an altered morphology and an impairment in sporulation. We identified several genes that could be responsible for the alterations in morphology in the Δ*cre1 *strain: a central position may be played by RAS1, a small GTPase that was repressed by CRE1 only at high growth rates but not by the growth rate itself. In the fission yeast *Schizosaccharomyces pombe*, Ras1 regulates two distinct pathways: one that controls mating through the Byr2-mitogen-activated protein kinase cascade and one that signals through Scd1-Cdc42 to maintain elongated cell morphology. In the filamentous fungi *Neurospora crassa *and *Aspergillus fumigatus*, the RAS1/RasA orthologue has been reported to regulate morphology, asexual development and cell wall integrity [39,40]. Also, *S. cerevisiae RAS1 *is regulated by glucose [41]. It is therefore possible that RAS1 is responsible for the phenotypic consequences of a *cre1 *loss-of-function, and the other genes may only indirectly be affected via *ras1*. In fact, three of the genes associated with morphology or sporulation (encoding PAG1, MedA and a phosphoproteoglycan, respectively) were repressed by CRE1 also at the high growth rate and the effect of CRE1 could therefore be via RAS1. Pag1 encodes a protein associated with protein kinase Cbk1p and that is required for cell morphogenesis and proliferation in *S. cerevisiae *[42]. *MedA *encodes an orthologue of *Fusarium oxysporum *REN1, *Aspergillus nidulans *MedA and *Magnaporthe grisea *ACR1 [43] that are transcription regulators involved in conidiogenesis, and whose loss-of-function leads to abnormal conidiophores and rod-shaped, conidium-like cells. Two other genes (encoding orthologues of the Blastomyces yeast phase specific extracellular protein BYS1, and the *A. fumigatus *cell wall protein PhiA) were affected by *cre1 *also at the high growth rate. This regulation under conditions where *ras1 *is repressed implies that their expression is independent of the CRE1 effects on *ras1 *and thus likely direct. Both genes contain a paired CRE1 binding motif in their promoter, which would support this assumption. Our data suggest that the interplay between CRE1 and RAS1 may be an important factor regulating developmental processes in *T. reesei.*

An investigation of the occurrence of the established CRE1 target sequence 5'-SYGGRG-3' in the regulated gene set confirmed its relevance to CCR, as 2 clusters out of the 6 that were regulated by CRE1 were also enriched in this CRE1 consensus site. In addition, an even higher enrichment was found for motif pairs biased toward CRERs (Cis Regulated Enriched Regions). These findings are in accordance with previous results showing that only a double CRE1 target is functionally *in vivo *[10,11]. In addition, we also identified an additional GC-rich (5'-SAGGGRGR-3') consensus to be overrepresented in CRE1 regulated promoters. This motif has not been previously described in filamentous fungi or any other organism, and we therefore do not know which proteins, if any, bind to it. However, a study on motifs in Mig1/Mig2/Mig3 regulated promoters in *S. cerevisiae *[5] detected a 5'-GGGAGG-3' motif, which is completely covered by the 5'-SAGGGRGR-3' sequence. The authors reasoned that the 5'-GGGAGG-3' motif may bind a transcription factor that regulates genes involved in phosphate metabolism. However, none of the genes of our study that show an enrichment of the 5'-SAGGGRGR-3' sequence in their 5' nontranslated sequences, are involved in phosphate metabolism. Notably, the presence or absence of the 5'-SYGGRG-3' motifs (or any other motif) in the promoters of regulated genes did not correlate with the way they are regulated by CRE1 (i.e. with a up- or a down-regulation).

## Conclusions

Carbon catabolite repression by CreA/CRE1 in filamentous fungi has mostly been studied in relation to the utilization of alternative carbon sources of either industrial interest (e.g. plant biomass components), or genetic model systems such as ethanol and proline catabolism [7,8,10,11], but these studies have so far not revealed the impact of this regulator on the physiology of the fungus in its natural environment. Here, we have identified new targets for *T. reesei *CRE1 and also dissected the dependence of their regulation on the rate of growth (equivalent to the nutritional condition) of the fungus. Noteworthy, a predominant effect of CCR seems to act at the transporter level and on the use of nitrogen substrates. Several genes that could explain morphological changes and sporulation behavior were also affected. Additionally, these growth-controlled environments also reflect some industrial conditions that can be used with fungi. The data and genes obtained will be a valuable basis for future attempts towards understanding the role of carbon nutrition for saprobic fungi. Finally, the strategy used in this paper may be useful also in further studies of other wide domain regulators in fungi.

## Methods

### Fungal strains and cultivation conditions

*H. jecorina *QM 9414 and the Δ*cre1 *strain derived from it (see below) were maintained on malt extract agar. Constant-mass, chemostat-type continuous cultivations were performed in a 2.5 l glass bioreactor with a working volume of 2 l, essentially as described earlier [31]. The feeding medium contained 3 g l^-1 ^glucose, a concentration low enough to make the culture carbon-limited. Steady-state of the cultures was established when no changes in biomass dry weight were observed in three successive samples taken over a period of three residence times (= the reciprocal value of the dilution rate). In D-glucose limited cultures, the steady-state biomass concentration was 1.46 ± 0.21 g l^-1^, irrespective of the dilution rate. The residual steady-state concentrations of D-glucose in the medium were 0.08-0.10 mM. The calculated growth yield (grams of biomass formed per gram of carbon source consumed) was between 46 and 49% for all cultures, which correlates well with our previous studies [34]. Two subsequently achieved, independent steady-states were sampled and analysed for each dilution rate and fungal strain.

### Construction of a *cre1 *knock-out strain of *T. reesei *QM 9414

A *cre1 *deletion vector was constructed by using the double joint PCR technique [44]. Primers used are given in Additional File 1, Table S6. Oligonucleotides cre5'F and Cre5'Rtailhph were used for the promoter region, cre3'R and Cre3'Ftailhph for the terminator region, and M1LHhph and M2LHhph for the hygromycin B expression cassette [45] amplification. The outside primers (cre5'F and cre3'R) were used for the amplification of the whole deletion fragment in a fusion PCR assay. The resulting fragment was subsequently cloned in pGEMT-Easy resulting in pΔcre1hph. The *cre1 *deletion fragment was released from plasmid pΔcre1hph by a *Not*I restriction digest. The respective 5.3 kb fragment was eluted with a QIAGEN Gel Extraction Kit (QIAGEN GmbH) and was used to transform protoplasts of the strain QM 9414 as described by Gruber *et al. *[46]. Transformants were selected on malt extract medium containing hygromycin (50 μg/ml). 40 transformants were selected from the transformation plates and transferred to small malt extract plates containing hygromycin (50 μg/ml). 25 transformants exhibited a stable phenotype and their respective spores were plated on malt extract medium containing hygromycin (50 μg/ml) and Triton X-100 (0.1%) to isolate single spore colonies. The *cre1 *loci of the six transformants and the parental strain QM 9414 as a control were amplified with the primers cre5'F and cre3'R using the following PCR conditions: 2 min of denaturation (94°C), were followed by 32 cycles of 45 s denaturation (94°C), 45 s of annealing (55°C), and 6 min of elongation (72°C), concluded by 7 min at 72°C. Genomic DNA of strain QM 9414 yielded a fragment of 4.3 kb, while homologous insertion of the deletion vector lead to an increase of the fragment size to 5.3 kb fragment in *cre1 *deleted strains. For the retransformation of the Δ*cre1 *strain the *cre1 *gene was amplified with oligonucleotides cre1retrafo1 and cre1retrafo2, which are located about 1500 bp upstream and 700 bp downstream of the coding region, respectively. The fragment was cloned into pGEM-T Easy (Promega). Transformation was performed with the *A. nidulans amdS *as marker [47]. Successful retransformation of the Δ*cre1 *strain with plasmid pcre1amdS was verified by PCR amplification of the *cre1 *gene and resulted in colonies, which showed a QM 9414 growth phenotype.

### Nucleic acid isolation and microarray hybridizations

Fungal mycelia were harvested by filtration, washed with distilled cold water, frozen and ground under liquid nitrogen. For extraction of genomic DNA, plasmid DNA and RNA, purification kits (Wizard Genomic DNA Purification Kit, PureYield Plasmid Midiprep System and SV Total RNA Isolation System, respectively, all from Promega) were used according to the manufacturer's protocol. Standard methods were used for electrophoresis, blotting and hybridization of nucleic acids. The microarray data and the related protocols are available at the GEO web site (http://www.ncbi.nlm.nih.gov/geo/) under accession number: GSE21072. Briefly, the fungal RNAs of each experiment were reverse-transcribed and labelled with Cy3 or Cy5 dye using the indirect labelling procedure and dye-switch on two biological replicates. We then hybridized 1 μg of labelled cDNA with the 1 × 244k *T. reesei *DNA chip manufactured by Agilent and designed using the Teloenn software as described previously [48]. Arrays were read using a GenePix 4000B scanner (Molecular Devices) and signals analysed by the GenePix Pro 6.1 software. Spots when the "align blocks" algorithm was not able to locate features on the slide were flagged "not found". A spot was labelled as "detectable" when the raw mean intensities were above the background. Data pretreatment was applied on each result file to discard GenePix flag and saturating spots. The data were normalized without background subtraction by the global Lowess method performed with the Goulphar software [49]. The background threshold was calculated by adding two standard deviations to the average intensity of all the "not found" features. For each probe the log2 hybridization ratio was linked to genome annotation coming from the JGI website. The final log2 ratio for each transcript was obtained by averaging the "detectable" hybridization values from all probes located inside the coding sequence on the matching strand. Transcripts with no or only one probe marked as "detectable" were discarded from further analysis. For the two biological replicates on each four experiments we applied on the pretreated results the linear modeling approach implemented by lmFit (using each replicate as independent variable) and the empirical Bayes statistics implemented by eBayes both from the limma R package [20]. We selected the list of statistically regulated genes with an adjusted p-value, using the Benjamini-Hochberg multiple test correction, lower than 0.05. Finally we kept as the most highly regulated targets only transcripts with a final log2 hybridization ratio greater than 2 or lower than -2.

### Real Time PCR quantification of *T. reesei *transcripts

DNase treated (DNase I, RNase free; Fermentas) RNA (5 μg), obtained from *T. reesei *QM 9414 and the delta-*cre1 *strain grown in chemostat cultures (*vide supra*), was reverse transcribed with the RevertAid™ First Strand cDNA Kit (Fermentas) according to the manufacturer's protocol with a combination of the provided oligo-dT and random hexamer primers. All real-time RT-PCR experiments were performed on a Bio-Rad (Hercules, CA) iCycler IQ. For the reaction the IQ SYBR Green Supermix (Bio-Rad, Hercules, CA) was prepared for 25 μl assays with standard MgCl2 concentration (3 mM) and a final primer concentration of 100 nM each. Primers used are given in Additional File 1, Table S7. All assays were carried out in 96-well plates, which were covered with optical tape. The amplification protocol consisted of an initial denaturation step (3 min at 95°C) followed by 40 cycles of denaturation (15 sec at 95°C), annealing (20 sec at 57°C) and elongation (10 sec at 72°C). Determination of the PCR efficiency was performed using triplicate reactions from a dilution series of cDNA (1, 0.1, 10^-2 ^10^-3^). Amplification efficiency was then calculated from the given slopes in the IQ5 Optical system Software v2.0. Expression ratios were calculated using REST^© ^Software [50].

### Biolog phenotype microarray experiments

Growth of *T. reesei *QM 9414 and two Δ*cre1 *independent transformed clones from it on 95 carbon sources was investigated using Biolog^® ^phenotype microarrays essentially as described by Druzhinina *et al. *[51]. Biomass concentration (OD750) was measured after 96 h of growth. One-way or main-effect analyses of variance (ANOVAs) were used to compare the growth of selected strains on individual carbon sources. Tukey's honest significant difference (Tukey HSD) test as implemented in STATISTICA 6.1 was used for post hoc comparisons to detect the contribution of each variable to the main effect of the F test resulting from the ANOVA. The significance of increased growth on selected carbon sources was tested by the Chi-square test. Only p-values < 0.05 were considered as significant.

### Cluster analysis of microarray results

Clustering analysis was done using the MultiExperiment Viewer software [52]. An expression matrix was built from all the genes sorted as strongly regulated for the four conditions we hybridized. From this matrix we selected the expression profiles corresponding to the main behaviour categories we expected which are: CRE1 repressed genes at high growth rate (GR), CRE1 induced genes at high GR, genes induced by CRE1 at low GR, genes repressed by CRE1 at low GR, genes repressed or induced by GR alone, CRE1 repressed genes alone and CRE1 induced genes alone. Then each of these gene expression groups was extended using the Pavlidis Template Matching method [53] fixating the absolute R threshold when all gene included in the group used as template are retrieved. Genes not classified in one of the groups where clustered using the Clustering Affinity Search Technique [54] procedure using the Pearson correlation coefficient and a 0.8 threshold. From the clusters obtained, two of them could be associated to already detected groups. Finally a hierarchical clustering was performed on each expression groups using Euclidian distance and the average linkage method. From this clustering each gene expression group was separated in two subclusters using the two more external nodes of each tree, resulting in clusters A to H and X.

### Gene identification and functional prediction

Genes were first identified according to their ID number. In the case of genes that were either poorly or not yet annotated, they were subjected to BLASTX at NCBI, and all hits with E < -50 retrieved and aligned. Proteins with >75% identity and over >90% of the amino acid sequence in other fungal taxa were considered to be their potential orthologues, and this hypothesis tested by phylogenetic analysis. Proteins that fell outside these criteria were termed "unknown proteins". Proteins with < 25% of identity and less then 75% of the size of the best hits were considered "unique". Identified proteins were categorized according to the Functional Catalogue (FunCat [55]) using the *T. reesei *genome database implemented in the Pedant server of the Munich Information Center for Protein Sequences [56]. For each of the FunCat categories selected we calculated for each gene cluster the enrichment ratio compared to the whole FunCat annotation and we used the hypergeometric distribution to compute the statistical p-value associated to this enrichment.

We performed a whole enrichment analysis for all the 171 annotated genes (among 250) detected as highly regulated targets using the Gene Ontology annotation of *T. reesei *from the JGI web site. We calculated the significance of the enrichment ratio using the hypergeometric distribution and the p-value where adjusted for multiple test using the Benjamini-Hochberg FDR correction. There are 4,977 genes annotated with at least one Gene Ontology term among the 9,129 genes from the *T. reesei *genome.

### Promoter sequence analysis

All analyses were performed with the RSAT software suite [24]. Promoter sequences were obtained using the "retrieve sequence" algorithm, with -1000 to -1 coordinates input and the "noORF" option unchecked. Motifs were searched using the "DNA Pattern Matching" algorithm, with the "prevent overlapping matches" parameter checked. For a given set of genes (any cluster, or whole genome), the total number of motifs found was collected and an average number of sites per gene was calculated. Finally this number was normalized to the average number of sites in the whole genome. Significance in mean values between cluster and whole genomes groups were assessed using a Student t-test with Microsoft Excel "ttest" function, using bidirectional and heteroscedastic (unequal variances) options. P-values of 0.1 and 0.05 were considered (and indicated in Table 1). For search of new regulation motifs, the "oligo analysis" and "Dyad Analysis" algorithms were used for each cluster or group of clusters. To ensure the selection of statistically relevant motifs, for each condition tested (Dyad, 5, 6, 7 or 8 words length), three different random gene groups of the same size were assessed in parallel (using the "random gene selection" algorithm). Only motifs with scores higher than the highest score obtained with the corresponding control set +0.5 were considered. This usually resulted in a 1.5 score selection threshold. Selected motifs were validated with enrichment in clusters and statistical tests described above.

## Authors' contributions

SLC, AM and TP designed and analysed the DNA arrays, EF and LK performed the chemostat cultivations, EK performed the morphological analysis, RL and BS did the Real Time PCR experiments, LA and ISD performed the Biolog Phenotype Microarray experiments. CPK designed the study, annotated the genes found and - together with TP, SLC and AM - wrote the paper. All authors read and approved the final manuscript.

## Supplementary Material

Additional file 1 **The CRE1 carbon catabolite repressor of the fungus *Trichoderma reesei*: a master regulator of carbon assimilation**. additional file 1 contains 4 figures (Figure S1 - S4) and 7 tables (Table S1-S7) that complement the results of the main paper: Figure S1: Morphological changes in *Trichoderma reesei Δcre1*. Figure S2: Effect of *Trichoderma reesei cre1*-knock out on biomass formation on different carbon sources. Figure S3: Growth of *T. reesei *wild-type and *cre1*-knock out on carbon sources whose utilization is CRE1-repressed. Figure S4: Distribution of gene profiles among experiments. Table S1: Average hyphal and cell wall width of *Trichoderma reesei *QM9414 and *Δcre1 *strains. Table S2: Transcripts and encoded proteins identified in this study. Table S3: Quantitative expression patterns determined by qRT-PCR of selected genes. Table S4: Enrichment analysis on FunCat categories. Table S5: Complete enrichment analysis with Gene Ontologies Table S6: Primers used for construction of the *T. reesei Δcre1 *strain. Table S7: Primers for Real Time quantification of selected genes. Click here for file
