# Supplementary material for: The CRE1 carbon catabolite repressor of the fungus Trichoderma reesei: a master regulator of carbon assimilation
Source: BMC Genomics. 2011 May 27;12:269. doi: 10.1186/1471-2164-12-269 (PMC3124439; doi:10.1186/1471-2164-12-269)
Supplement: Additional file 1 — The CRE1 carbon catabolite repressor of the fungus Trichoderma reesei: a master regulator of carbon assimilation. additional file 1 contains 4 figures (Figure S1 - S4) and 7 tables (Table S1-S7) that complement the results of the main paper: Figure S1: Morphological changes in Trichoderma reesei Δcre1. Figure S2: Effect of Trichoderma reesei cre1-knock out on biomass formation on different carbon sources. Figure S3: Growth of T. reesei wild-type and cre1-knock out on carbon sources whose utilization is CRE1-repressed. Figure S4: Distribution of gene profiles among experiments. Table S1: Average hyphal and cell wall width of Trichoderma reesei QM9414 and Δcre1 strains. Table S2: Transcripts and encoded proteins identified in this study. Table S3: Quantitative expression patterns determined by qRT-PCR of selected genes. Table S4: Enrichment analysis on FunCat categories. Table S5: Complete enrichment analysis with Gene Ontologies Table S6: Primers used for construction of the T. reesei Δcre1 strain. Table S7: Primers for Real Time quantification of selected genes. [file 1471-2164-12-269-S1.DOC]

**The CRE1 carbon catabolite repressor of the fungus *Trichoderma reesei*:
a master regulator of carbon assimilation**

**ADDITIONAL FILE 1**

**Figure S1:** Morphological changes in *Trichoderma reesei* *Δcre1*.

Morphology of the hyphae of *T. reesei* QM 9414 (A, C) and its *Δcre1* mutant strain (B, D). Microscopy was performed with a Zeiss AxioImager equipped with AxioCam MRc5 camera and AxioVision AC image analyser system. Pictures were taken either with 20X "Plan‐ Apochromat" objective and DICK filter, or with 100x „EC Plan‐Neofluar” with oil immersion.

The parent strain differed from the *Δcre1* strain of *T. reesei* in the following morphological attributes: (i) it developed longer hyphae; (ii) the *Δcre1* mutant displayed more robust hyphae, containing a considerably thickened cell wall (see also Supplementary Table 1); and (iii) the *Δcre1* mutant exhibited less septa in its hyphae. All data were obtained on malt extract agar.

**Figure S2:** Effect of *Trichoderma reesei* *cre1*-knock out on biomass formation on different carbon sources .

Growth of *T. reesei* QM 9414 and two *Δcre1* clones on selected carbon sources. Experiments were performed using Biolog® phenotype microarrays essentially as described by Druzhinina *et al.* (2006). Biomass concentration (OD_750_) is represented after 96 h of growth on 95 different carbon sources. QM 9414 is given by the black line, the two *Δcre1* strains by red and blue lines, respectively. Red arrows pinpoint carbon sources resulting in a significantly reduced growth in the *Δcre1* strains. Data are means of 3 biological replicates, which did not differ more than ± 12%.

**Figure S3:** Growth of *T. reesei* wild-type and *cre1*-knock out on carbon sources whose utilization is CRE1‐repressed.

Growth curves of *T. reesei* QM 9414 and two *Δcre1* independent clones on selected carbon sources whose utilization is CRE1‐repressed obtained in 96h of incubation time. Experiments were performed using Biolog® phenotype microarrays essentially as described by Druzhinina et *al.* (2006). The lilac line specifies QM 9414; the blue and red lines specify the two *Δcre1* strains. A, D‐galactose; B, L‐sorbose; C, D‐xylose; D, palatinose (6‐O‐α‐D‐Glucopyranosyl‐D‐
fructoside); E, maltose; F, stachyose (β‐D‐Fructofuranosyl‐O‐α‐D‐ galactopyranosyl‐(1→6)‐ O‐α‐D‐ galactopyranosyl‐(1→6)‐α‐D‐ glucopyranoside); G, xylitol; H, ribitol; J, glucuronamide. Data are means of 3 biological replicates, which did not differ more than ± 12%.

**Figure S4:** Distribution of gene profiles among experiments.


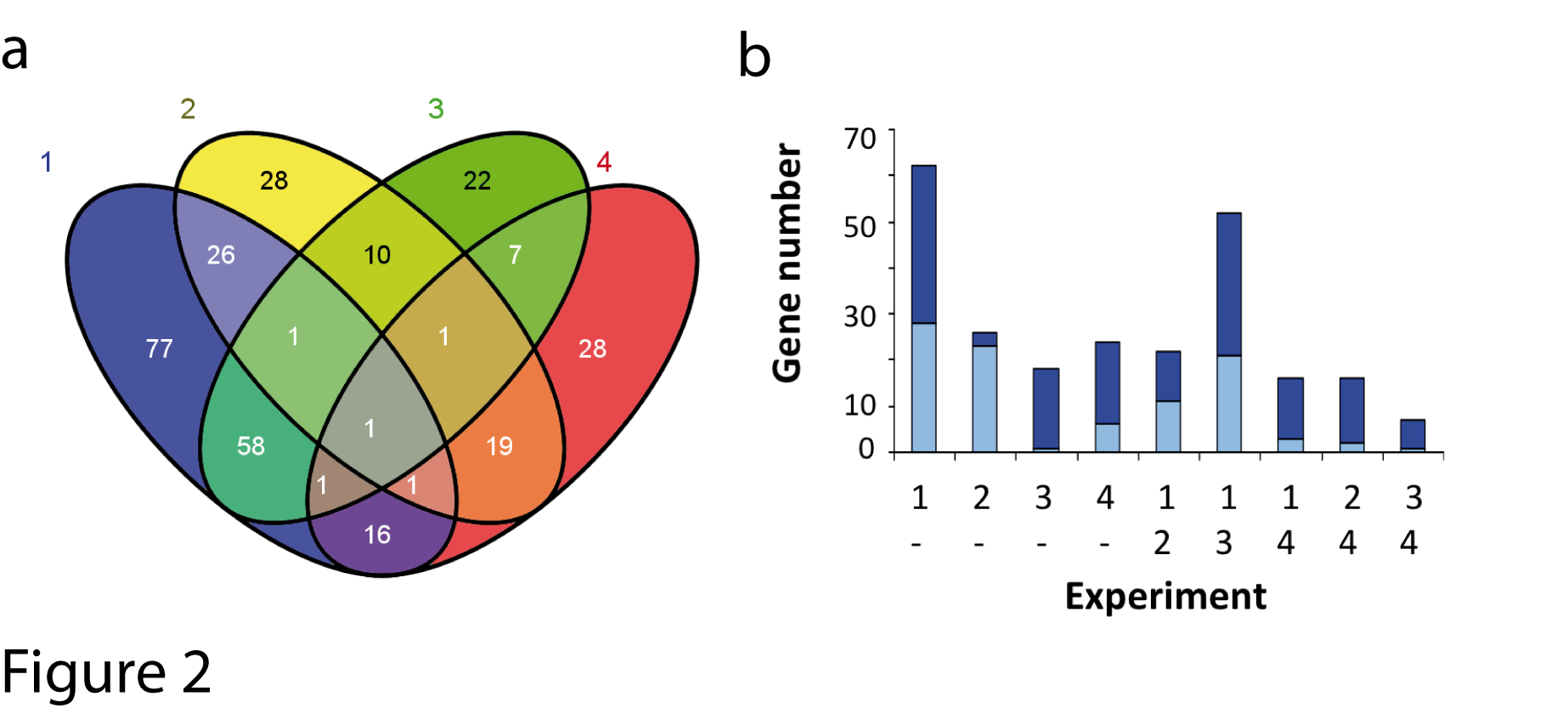


(**a**) VENN diagram showing the distribution of genes found to be the most highly regulated between the 4 experiments outlined in Figure 1 (indicated by numbers). (**b**) Distribution of upregulated (dark blue) and downregulated genes (light blue) in experiments 1-4, and combinations of them (indicated by numbers on the x-axis). In the combined experiments given, regulation occurred in the same direction. The combination of experiment 2 and 3 is not shown, as all genes were downregulated in experiment 2 but upregulated in experiment 3.

**Table S1:** Average hyphal and cell wall width of *Trichoderma reesei* QM9414 and *Δcre1* strains. Data are given as the average of 50 measures. Significant differences are observed for these both physiological features (S. D.: Standard Deviation).

| Strain | Hyphal width  (S.D.)  (µm) | Cell wall width  (S.D.)  (µm) |
| --- | --- | --- |
| QM9414 | **2.277**  ± 0.2628 | **0.3981**  ± 0.0449 |
| *Δcre1* | **2.816**  ±0.2951 | **0.5412**  ± 0.0693 |

**Table S2:** Transcripts and encoded proteins identified in this study. Numbers in column headers refer to the conditions as specified in the experimental design (Figure 1). The four penultimate columns are expressed as log_2_ fold change.

| **protein ID** | **Function** | **category** | **1** | **2** | **3** | **4** | **cluster** |
| --- | --- | --- | --- | --- | --- | --- | --- |
| 1751 | FAD monooxygenase | Energy; Protein with binding function or cofactor requirement; | -3.102 | -1.567 | -0.847 | 0.244 | X |
| 1885 | glucamylase with starch binding domain | Metabolism; C-compound and carbohydrate metabolism; | 2.009 | -0.29 | 2.399 | -0.157 | E |
| 1925 | fatty acid hydroxylase | Metabolism; Lipid, fatty acid and isoprenoid metabolism; | -2.073 | -2.014 | 0.437 | 0.874 | B |
| 3327 | NADH:flavin oxidoreductase/NADH oxidase | Energy; Protein with binding function or cofactor requirement; | -2.289 | NA | -1.9 | 0.102 | F |
| 3363 | hypothetical protein with 9 transmembrane domains | Hypothetical protein; | NA | -1.541 | 2.531 | 1.769 | C |
| 4950 | urea transporter | Cellular transport, transport facilitation and transport routes; | -0.092 | -0.759 | 1.755 | 2.284 | C |
| 4999 | P450 monooxygenase | Metabolism; Secondary metabolism; | 2.63 | 1.276 | 2.376 | 1.596 | E |
| 21412 | secreted protein, hypothetical | Hypothetical protein; | -0.473 | -2.045 | -0.274 | -2.01 | G |
| 21725 | GH20 N-acetyl-glucosaminidase NAG1 | Metabolism; C-compound and carbohydrate metabolism; | 2.101 | 0.742 | 0.608 | -0.852 | D |
| 21758 | isocitrate lyase | Energy; | 1.633 | -0.489 | 2.868 | 0.855 | E |
| 21908 | fatty acid elongase | Metabolism; Lipid, fatty acid and isoprenoid metabolism; | -2.459 | -1.654 | -1.519 | -1.055 | X |
| 21960 | phospholipase C | Cellular communication, signal transduction mechanism; | 2.085 | 0.277 | 0.547 | -1.337 | D |
| 22459 | putative carboxypeptidase A | Protein fate; | 2.561 | 2.671 | NA | 0.282 | A |
| 22912 | glucose transporter HXT1 | Cellular transport, transport facilitation and transport routes; | 4.852 | 2.866 | 1.726 | -0.971 | A |
| 23171 | peptaibol synthase | Metabolism; Secondary metabolism; | 2.658 | -1.359 | 1.231 | -3.28 | E |
| 23228 | hypothetical protein Fuf1479 | Hypothetical protein; | 3.214 | 2.806 | -0.215 | -1.251 | A |
| 23382 | aldehyde reductase AKR7 | Metabolism; Metabolism general; | -5.146 | NA | -4.473 | NA | F |
| 23415 | amino acid permease Dip5 | Metabolism; Amino acid metabolism; Cellular transport, transport facilitation and transport routes; | 1.496 | -0.122 | 2.697 | 1.093 | E |
| 27992 | PTH11-type G-coupled receptor | Cellular communication, signal transduction mechanism; | 2.612 | -0.201 | 2.123 | -0.715 | E |
| 30759 | protein of zinc containing alcohol dehydrogenase superfamily | Energy; | -3.835 | 0.307 | -3.465 | -0.249 | F |
| 32712 | NACHT domain protein with ankyrin repeats | Hypothetical protein; | 3.039 | 1.742 | 3.073 | 1.713 | E |
| 37525 | G-coupled receptor GprK-type | Cellular communication, signal transduction mechanism; | 2.251 | 0.555 | 1.048 | -0.832 | D |
| 38812 | iron transporter | Cellular transport, transport facilitation and transport routes; | 2.408 | 0.912 | NA | -1.873 | D |
| 39637 | C4-dicarboxylate transporter | Cellular transport, transport facilitation and transport routes; | -0.327 | 1.178 | 1.666 | 3.261 | X |
| 41895 | glutathione-S-transferase | Cell rescue, defense and virulence; | -2.627 | -2.433 | -0.41 | -0.613 | B |
| 43671 | ammonium permease MEA1 | Cellular transport, transport facilitation and transport routes; | -3.49 | -0.017 | -0.063 | 4.003 | X |
| 43701 | MSF multidrug transporter | Cellular transport, transport facilitation and transport routes; | -4.594 | 0.197 | -5.722 | -2.668 | F |
| 44175 | lactate pyruvate transporter | Cellular transport, transport facilitation and transport routes; | 2.428 | 0.594 | 1.152 | -0.54 | D |
| 44230 | GTP-binding protein EsdC | Cellular communication, signal transduction mechanism; | 2.669 | 1.379 | -0.065 | -1.598 | D |
| 44747 | SNF2 family helicase | Cell cycle and DNA processing; Transcription; | 4.214 | 0.716 | 3.2 | -0.852 | E |
| 44967 | hypothetical protein, conserved | Hypothetical protein; | -2.72 | -2.626 | -0.25 | -0.176 | B |
| 44987 | ERG25 methylsterol desaturase | Metabolism; Lipid, fatty acid and isoprenoid metabolism; | -1.503 | -2.228 | 0.322 | -0.083 | B |
| 45138 | sulfite reductase, ß-subunit | Metabolism; Nitrogen and sulfur metabolism; Cellular transport, transport facilitation and transport routes; | -0.125 | 2.119 | -0.227 | 1.983 | H |
| 45250 | oleate-delta12-desaturase | Metabolism; Lipid, fatty acid and isoprenoid metabolism; | -1.956 | -2.934 | 0.073 | -0.963 | B |
| 45445 | tyrosinase | Metabolism; Secondary metabolism; Protein with binding function or cofactor requirement; | 2.53 | -0.032 | 2.385 | -0.211 | E |
| 45717 | GH47 α-1,2-mannosidase | Metabolism; C-compound and carbohydrate metabolism; | 2.3 | 2.044 | -0.165 | -0.655 | A |
| 46209 | SAM-methyltransferase | Metabolism; Metabolism general; Protein with binding function or cofactor requirement; | NA | 1.484 | 0.258 | 2.21 | H |
| 46794 | MSF peptide transporter | Cellular transport, transport facilitation and transport routes; | 1.571 | -0.191 | 3.069 | 1.413 | E |
| 47066 | candidate sulfate adenylyltransferase | Cellular transport, transport facilitation and transport routes; | 0.151 | 1.567 | 0.664 | 2.305 | H |
| 47710 | monosaccharide transporter | Cellular transport, transport facilitation and transport routes; | 3.649 | -1.389 | 4.371 | -0.608 | E |
| 48211 | hypothetical conserved protein, intracellular | Hypothetical protein; | 0.288 | 1.685 | 1.691 | 3.351 | X |
| 49205 | cytochrome C peroxidase | Cell rescue, defense and virulence; | -2.117 | -1.476 | -0.409 | 0.669 | B |
| 49274 | GH16 ß-1,3/4-glucanase | Metabolism; C-compound and carbohydrate metabolism; | -2.728 | -1.565 | -1.549 | -0.101 | X |
| 49589 | unique protein | Unique protein; | -2.137 | -1.848 | -1.098 | -0.663 | B |
| 49898 | glucosamine-6-phosphate isomerase | Metabolism; C-compound and carbohydrate metabolism; | 3.169 | 2.196 | 0.676 | -0.415 | A |
| 49970 | H+ nucleoside cotransporter | Cellular transport, transport facilitation and transport routes; | -0.074 | -1.356 | 2.746 | 1.679 | C |
| 50104 | phosphatidyl synthase | Metabolism; Lipid, fatty acid and isoprenoid metabolism; | NA | -2.211 | 2.113 | 0.575 | G |
| 50212 | distantly related to glycosyltransferases | Metabolism; C-compound and carbohydrate metabolism; | -1.661 | -4.402 | 1.623 | -1.051 | G |
| 50215 | GH16 candidate ß-glycosidase | Metabolism; C-compound and carbohydrate metabolism; | -2.049 | -3.941 | 0.943 | -0.729 | B |
| 50618 | MSF transporter | Cellular transport, transport facilitation and transport routes; | 2.719 | 1.275 | 1.293 | 0.165 | D |
| 51110 | IndA1 amino acid transporter | Cellular transport, transport facilitation and transport routes; | 2.153 | -0.645 | 3.93 | 0.864 | E |
| 51650 | hypothetical protein | Hypothetical protein; | 0.855 | -0.81 | 2.527 | 0.751 | E |
| 51893 | putative peroxidase/hem containing | Metabolism; Metabolism general; Protein with binding function or cofactor requirement; | 2.796 | 0.794 | 1.945 | -0.253 | E |
| 52055 | mitochondrial NADP-dependent isocitrate dehydrogenase | Energy; | -0.815 | 1.251 | -0.236 | 2.203 | H |
| 52315 | Ctr copper transporter | Cellular transport, transport facilitation and transport routes; | -0.52 | -2.001 | 0.473 | -1.402 | G |
| 54198 | hypothetical protein, conserved | Hypothetical protein; | -0.031 | -2.072 | 0.869 | -1.337 | G |
| 54352 | hypothetical protain, conserved | Hypothetical protein; | NA | 2.457 | NA | 1.25 | H |
| 54511 | hypothetical protein, contains BTB/POZ domain involved in protein binding | Hypothetical protein; | 2.1 | 0.757 | 0.623 | -0.861 | D |
| 54667 | acyl-CoA synthetase | Metabolism; Lipid, fatty acid and isoprenoid metabolism; | 2.469 | 1.493 | 2.267 | 0.75 | E |
| 54768 | hypothetical protein | Hypothetical protein; | 2.299 | -0.612 | 1.471 | -1.201 | E |
| 55272 | hypoothetical protein | Hypothetical protein; | 5.813 | 0.321 | 6.216 | -0.097 | E |
| 55319 | GH54, L-α-arabinofuranosidase | Metabolism; C-compound and carbohydrate metabolism; | 3.445 | 1.434 | 0.331 | -1.782 | D |
| 55443 | hypothetical protein, secreted | Hypothetical protein; | 0.924 | -2.275 | 2.947 | 0.374 | G |
| 55886 | GH16 ß-glycosidase (GPI anchor) | Metabolism; C-compound and carbohydrate metabolism; | NA | 2.034 | NA | 2.701 | H |
| 55990 | hypothetical protein | Hypothetical protein; | 2.699 | 0.018 | NA | -2.555 | D |
| 57204 | lipase, secreted | Metabolism; Lipid, fatty acid and isoprenoid metabolism; Extracellular metabolism; | NA | 1.886 | NA | 2.275 | H |
| 57749 | MSF transporter | Cellular transport, transport facilitation and transport routes; | 1.625 | -0.355 | NA | -2.914 | D |
| 58264 | putative paxU orthologue involved in indole-terpene biosynthesis | Metabolism; Secondary metabolism; | -2.117 | -0.743 | -0.849 | 0.419 | X |
| 58282 | CE9 esterase | Metabolism; C-compound and carbohydrate metabolism; | 2.869 | -0.092 | 3.445 | 0.579 | E |
| 58356 | glycerol kinase | Metabolism; C-compound and carbohydrate metabolism; | 2.379 | 0.856 | NA | -1.717 | D |
| 59014 | ABC multidrug transporter | Cellular transport, transport facilitation and transport routes; Cell rescue, defense and virulence; | -2.544 | -0.375 | -2.036 | 0.264 | F |
| 59151 | BYS1 domain protein (=Blastomyces yeast-phase-specific protein) secreted | Classification not yet clear-cut; | 0.804 | -1.955 | 2.349 | 0.145 | G |
| 59173 | MSF transporter | Cellular transport, transport facilitation and transport routes; | NA | 1.654 | 0.184 | 2.159 | H |
| 60489 | CE5 cutinase | Metabolism; C-compound and carbohydrate metabolism; | 2.22 | 1.824 | NA | -1.844 | D |
| 60616 | integral membrane protein | Hypothetical protein; | -2.972 | -0.45 | -2.362 | 0.363 | F |
| 60635 | GH92 α-1,2-mannosidase | Metabolism; C-compound and carbohydrate metabolism; | 2.124 | 0.684 | NA | -2.383 | D |
| 60847 | ATP synthase subunit 9, proteolipid P2 | Energy; | -4.492 | -0.778 | -1.201 | 2.527 | X |
| 62100 | Hsp30 | Protein fate; Cell rescue, defense and virulence; | 0.438 | -1.882 | 2.078 | -0.424 | G |
| 62172 | amino acid permease | Cellular transport, transport facilitation and transport routes; | NA | -0.929 | 2.765 | 2.564 | C |
| 64874 | MSF toxin efflux pump | Cellular transport, transport facilitation and transport routes; | 1.69 | -1.269 | 2.527 | 0.06 | E |
| 64959 | phosphatidyl synthase | Metabolism; Lipid, fatty acid and isoprenoid metabolism; | -2.234 | -0.43 | -0.581 | 1.661 | X |
| 65191 | maltose permease | Cellular transport, transport facilitation and transport routes; | 1.826 | 0.025 | 2.502 | 0.661 | E |
| 65406 | GH16, glucanosyltransferase MWG2 (orthologue of A. fumgatus Crf1) | Metabolism; C-compound and carbohydrate metabolism; | 0.262 | -2.338 | 1.432 | -1.215 | G |
| 65410 | Phosphoadenosine phosphosulfate reductase | Metabolism; Amino acid metabolism; | -0.14 | 3.455 | -0.548 | 3.56 | H |
| 65522 | unique protein | Unique protein; | -1.033 | -2.348 | 0.714 | -0.09 | B |
| 65741 | hypothetical transmembrane protein | Hypothetical protein; | 2.682 | 0.807 | 1.034 | -1.001 | D |
| 66041 | hypothetical protein | Hypothetical protein; | -2.147 | -1.116 | -1.389 | NA | F |
| 67275 | RAS1 | Protein activity regulation; Cellular communication, signal transduction mechanism; | 1.838 | -0.341 | 2.221 | -0.027 | E |
| 68608 | Thiazole biosynthetic enzyme (orthologue of Stress-inducible protein sti35) | Cell rescue, defense and virulence; | -4.435 | -3.889 | -0.748 | -0.262 | B |
| 68812 | MSF sugar transporter | Cellular transport, transport facilitation and transport routes; | 2.623 | 1.496 | 2.358 | 1.275 | E |
| 68924 | putative cyclopropane/fatty acid synthase | Metabolism; Lipid, fatty acid and isoprenoid metabolism; | 2.348 | 2.066 | NA | 0.669 | A |
| 69245 | GH2 ß-mannosidase | Metabolism; C-compound and carbohydrate metabolism; | 2.261 | 0.923 | NA | -1.825 | D |
| 70520 | short chain dehydrogenase/reductase | Metabolism; Metabolism general; Protein with binding function or cofactor requirement; | NA | 0.848 | -0.963 | 2.023 | H |
| 70860 | allantoate permease | Metabolism; Nitrogen and sulfur metabolism; Cellular transport, transport facilitation and transport routes; | 0.288 | 0.185 | 2.229 | 2.319 | C |
| 71532 | GH71 α-1,3-glucanase | Metabolism; C-compound and carbohydrate metabolism; | 1.265 | -2.698 | 3.062 | -0.244 | G |
| 72091 | hypothetical protein | Hypothetical protein; | 2.193 | 0.078 | NA | -0.321 | D |
| 72379 | conidiospore surface protein CMP1 | Biogenesis of cellular components; | NA | 5.72 | NA | 6.19 | H |
| 72922 | GABA permease | Metabolism; Amino acid metabolism; | -2.434 | -1.335 | 0.46 | 1.815 | C |
| 73179 | GH95 α-fucosidase | Metabolism; C-compound and carbohydrate metabolism; | 2.426 | 0.574 | NA | -3.014 | D |
| 73618 | polyketide synthase | Metabolism; Secondary metabolism; | -4.181 | -0.202 | -3.623 | NA | F |
| 73621 | polyketide synthase | Metabolism; Secondary metabolism; | -4.415 | -0.307 | -3.929 | NA | F |
| 73623 | FAD-monooxygenase | Energy; Protein with binding function or cofactor requirement; | -3.494 | -0.111 | -2.821 | 0.056 | F |
| 73631 | isoamyl alcohol oxidase | Metabolism; Secondary metabolism; | -4.418 | NA | -4.09 | NA | F |
| 73818 | catalase C | Energy; | 1.889 | -1.623 | 3.608 | 0.023 | E |
| 74060 | hypothetical protein with similarity to *Aspergillus* allergen | Hypothetical protein; | -2.696 | -1.899 | -1.22 | -0.758 | B |
| 74194 | L-xylulose reductase LXR1 | Metabolism; C-compound and carbohydrate metabolism; | -2.326 | -0.438 | -2.011 | -0.231 | F |
| 74198 | GH92 α-1,2-mannosidase | Metabolism; C-compound and carbohydrate metabolism; | 2.513 | NA | NA | -3.79 | D |
| 74278 | Pyridine nucleotide-disulphide oxidoreductase, class-II | Metabolism; Nucleotide metabolism; | -2.349 | -0.923 | -2.194 | -0.399 | F |
| 74282 | hypothetical cysteine-rich protein | Hypothetical protein; | 4.55 | -2.113 | 3.228 | -4.398 | E |
| 74580 | hypothetical transmembrane protein | Hypothetical protein; | 2.996 | -1.783 | 4.06 | -0.771 | E |
| 74807 | hypothetical glycosyl hydrolase, not aligned with a GH family | Metabolism; C-compound and carbohydrate metabolism; | 2.509 | 1.821 | 0.722 | 0.133 | A |
| 74854 | stearic acid desaturase Sde1 | Metabolism; Lipid, fatty acid and isoprenoid metabolism; | -2.23 | -3.066 | 0.439 | -0.319 | B |
| 75383 | short chain dehydrogenase/reductase | Metabolism; Metabolism general; Protein with binding function or cofactor requirement; | -0.384 | -0.101 | 1.187 | 2.133 | C |
| 76215 | sulfide:quinone oxidoreductase | Energy; | -0.476 | 1.101 | 1.019 | 3.095 | X |
| 76218 | protein of zinc containing alcohol dehydrogenase superfamily | Metabolism; Secondary metabolism; Protein with binding function or cofactor requirement; | -0.929 | -2.102 | 0.663 | 0.098 | B |
| 76238 | plasma membrane H+ ATPase | Cellular transport, transport facilitation and transport routes; | -2.957 | 0.126 | -1.548 | 1.626 | F |
| 76288 | short chain dehydrogenase/reductase | Metabolism; Metabolism general; Protein with binding function or cofactor requirement; | -0.764 | 1.18 | -0.374 | 2.327 | H |
| 76381 | urate oxidase | Metabolism; Secondary metabolism; | -1.282 | -2.149 | 0.978 | 0.286 | B |
| 76633 | MDR transporter | Cellular transport, transport facilitation and transport routes; | -0.05 | 2.057 | -0.964 | 1.694 | H |
| 76722 | flavohemoglobin | Energy; | -2.299 | -1.748 | -0.448 | NA | B |
| 76763 | PTH11-type G-coupled receptor | Cellular communication, signal transduction mechanism; | -2.366 | -0.225 | -2.298 | 0.138 | F |
| 76766 | xanthine/uracil permease | Cellular transport, transport facilitation and transport routes; | -1.536 | -1.225 | 1.487 | 2.137 | C |
| 76852 | GH2 glycoside hydrolase | Metabolism; C-compound and carbohydrate metabolism; | 1.879 | NA | NA | -2.613 | D |
| 76897 | MSF transporter | Cellular transport, transport facilitation and transport routes; | 2.697 | 0.9 | 1.824 | -0.317 | E |
| 77093 | acid sphingomyelin phosphodiesterase (probably vacuolar) | Metabolism; Lipid, fatty acid and isoprenoid metabolism; | 1.73 | -0.481 | -0.114 | -2.864 | D |
| 77481 | D-xylulose 5-phosphate/D-fructose 6-phosphate phosphoketolase | Energy; | -2.806 | -2.651 | -0.024 | 0.118 | B |
| 77512 | cytochrome P450 | Metabolism; Secondary metabolism; | 2.246 | 1.673 | NA | 0.204 | A |
| 77547 | GT1 glycosyltransferase | Metabolism; C-compound and carbohydrate metabolism; | NA | -2.396 | 0.798 | NA | G |
| 78683 | aldehyde dehydrogenase | Energy; | 2.019 | 0.57 | 0.807 | -0.66 | D |
| 78688 | Hypothetical protein with a heat shock factor-type DNA-binding domain | Cell rescue, defense and virulence; | -0.798 | 0.486 | 0.799 | 2.566 | X |
| 78970 | mitochondrial (phosphate) carrier | Energy; | -2.462 | -0.715 | -1.354 | 0.889 | F |
| 79671 | N-acetyl-glucosamine-6-phosphate deacetylase | Metabolism; C-compound and carbohydrate metabolism; | 2.297 | 0.991 | NA | -0.572 | A |
| 79677 | N-acetyl-glucosamine kinase | Metabolism; C-compound and carbohydrate metabolism; | 2.111 | 1.223 | NA | -0.739 | A |
| 79741 | ER-bound Farnesyl-diphosphate farnesyltransferase | Protein fate; | -0.658 | 3.335 | -0.326 | 3.913 | H |
| 79816 | hypothetical secreted protein | Hypothetical protein; | 2.342 | 0.465 | 0.053 | -3.891 | D |
| 80019 | short-chain dehydrogenase/reductase | Metabolism; Metabolism general; Protein with binding function or cofactor requirement; | 2.39 | 0.766 | 0.401 | -0.664 | D |
| 80026 | MSF permease | Cellular transport, transport facilitation and transport routes; | 0.854 | -2.631 | 2.23 | -1.683 | E |
| 80086 | MSF peptide transporter | Cellular transport, transport facilitation and transport routes; | NA | -2.374 | 2.549 | 1.057 | C |
| 80149 | hypothetical protein, conserved | Hypothetical protein; | 2.006 | 0.571 | 2.256 | 0.726 | E |
| 80659 | alcohol oxidase AOX1 | Metabolism; Metabolism general; | 2.633 | NA | NA | -3.664 | D |
| 80833 | N-acetylglucosamine permease | Metabolism; C-compound and carbohydrate metabolism; | 3.445 | 3.095 | NA | -1.144 | A |
| 80863 | GH18 endochitinase CHI18-5 | Cellular transport, transport facilitation and transport routes; | 4.071 | -0.69 | 4.615 | -0.296 | E |
| 80879 | H+/oligopeptide transporter | Cellular transport, transport facilitation and transport routes; | -3.313 | -1.331 | -1.721 | 1.104 | F |
| 80920 | alcohol dehdrogenase ADH1 | Metabolism; Secondary metabolism; | -1.511 | -2.455 | 0.201 | -1.07 | B |
| 80980 | porphyromonas-type peptidyl arginine deiminase | Metabolism; Amino acid metabolism; | 5.239 | 1.585 | 4.652 | 0.732 | E |
| 81004 | aspartate protease, secreted | Protein fate; | NA | 2.439 | -0.3 | 2.506 | H |
| 81022 | allantoate permease | Cellular transport, transport facilitation and transport routes; | NA | -1.441 | 3.252 | 2.201 | C |
| 81082 | aquaglyceroporin | Cellular transport, transport facilitation and transport routes; | NA | -3.819 | 3.167 | NA | G |
| 81149 | aquaglyceroporin | Cellular transport, transport facilitation and transport routes; | 2.173 | -0.413 | 1.018 | -2.029 | E |
| 81383 | G-protein coupled receptor, GprK-like, | Cellular communication, signal transduction mechanism; | 3.569 | 1.65 | 2.356 | 0.145 | E |
| 81525 | isoflavone reductase | Metabolism; Secondary metabolism; | -2.183 | -0.071 | -1.903 | -0.024 | F |
| 81576 | hypothetical assimilatory sulfite reductase, alpha subunit | Metabolism; Nitrogen and sulfur metabolism; | -1.024 | 3.903 | -1.552 | 3.416 | H |
| 81598 | GH18 endochitinase CHI18-7 | Metabolism; C-compound and carbohydrate metabolism; | -2.11 | -0.807 | -1.606 | -0.334 | F |
| 81690 | orthlogue of *Aspergillus nidulans* CreD | Cellular communication, signal transduction mechanism; | -2.183 | -1.355 | -0.709 | 0.542 | X |
| 81778 | glutaminase A | Metabolism; Amino acid metabolism; | 1.805 | -0.01 | NA | -2.432 | D |
| 81979 | glutathione-S-transferase | Cell rescue, defense and virulence; | -2.258 | 0.36 | -1.975 | 0.941 | F |
| 82026 | trans-aconitate methyltransferase | Metabolism; Metabolism general; | NA | 4.152 | NA | 5.325 | H |
| 82032 | hypothetical protein | Hypothetical protein; | -3.934 | -3.825 | -0.42 | -0.233 | B |
| 82095 | high affinity ammonium transporter | Metabolism; Amino acid metabolism; Cellular transport, transport facilitation and transport routes; | -1.801 | -1.623 | 3.913 | 4.612 | C |
| 82204 | high affinity ammonium transporter | Metabolism; Amino acid metabolism; Cellular transport, transport facilitation and transport routes; | -2.324 | 2.408 | -2.377 | 2.806 | F |
| 82208 | polyketide synthase | Metabolism; Secondary metabolism; | NA | 3.697 | 1.493 | 5.438 | H |
| 82235 | GH31 α-glucosidase | Metabolism; C-compound and carbohydrate metabolism; | 2.343 | -0.872 | 1.227 | -2.147 | E |
| 102497 | Zn2Cys6 transcription factor | Transcription; Protein activity regulation; | -2.614 | 0.217 | -3.466 | -0.823 | F |
| 102499 | Zn2Cys6-transcription factor | Transcription; Protein activity regulation; | -2.424 | -0.346 | -2.099 | NA | F |
| 103015 | hypothetical protein | Hypothetical protein; | -3.104 | NA | -3.325 | NA | F |
| 103179 | unique protein | Unique protein; | NA | -2.926 | 4.849 | 0.678 | G |
| 104322 | unique protein | Unique protein; | 2.22 | 1.241 | NA | -0.02 | A |
| 105106 | hypothetical protein | Hypothetical protein; | 0.912 | -2.107 | 1.526 | -0.848 | G |
| 105224 | hypothetical membrane protein | Hypothetical protein; | NA | -2.087 | NA | NA | G |
| 105313 | unique protein | Unique protein; | -1.394 | -2.503 | 1.986 | -0.876 | G |
| 105752 | C4 dicarboxylate transporter | Cellular transport, transport facilitation and transport routes; | -3.547 | 0.411 | -3.091 | NA | F |
| 106248 | monosaccharide transporter | Metabolism; C-compound and carbohydrate metabolism; Cellular transport, transport facilitation and transport routes; | 2.781 | 1.685 | NA | -0.622 | A |
| 106556 | hypothetical protein | Hypothetical protein; | -5.06 | -0.155 | -5.095 | NA | F |
| 107055 | hypothetical protein | Hypothetical protein; | 0.831 | 1.173 | 0.939 | 2.173 | X |
| 107639 | hypothetical protein | Hypothetical protein; | -2.593 | -0.003 | -2.29 | 0.356 | F |
| 107881 | hypothetical protein | Hypothetical protein; | NA | -2.496 | 1.896 | 0.53 | G |
| 107960 | hypothetical protein with WSC-carbohydrate binding domain, secreted | Hypothetical protein; | NA | 2.117 | 0.478 | 2.947 | H |
| 108143 | unique protein | Unique protein; | 2.291 | NA | NA | -4.145 | D |
| 108357 | hypothetical protein | Hypothetical protein; | 4.071 | 0.511 | 4.2 | 0.43 | E |
| 108586 | hypothetical protein | Hypothetical protein; | 3.041 | 1.403 | NA | -0.152 | A |
| 108642 | hypothetical secreted protein | Hypothetical protein; | -0.109 | 1.742 | -0.176 | 2.063 | H |
| 108914 | hypothetical protein | Hypothetical protein; | -1.591 | -2.635 | -0.469 | -2.006 | G |
| 109234 | putative D-aminopeptidase | Protein fate; | NA | -2.051 | 1.41 | 0.252 | G |
| 110620 | hypothetical protein | Hypothetical protein; | -2.155 | -1.552 | -0.941 | -0.191 | B |
| 111082 | putative glutathione S transferase | Cell rescue, defense and virulence; | -2.216 | -3.368 | -0.45 | -1.763 | B |
| 111750 | ferric reductase | Metabolism; Metabolism general; Protein with binding function or cofactor requirement; Cellular transport, transport facilitation and transport routes; | 0.326 | -2.256 | 1.819 | -1.065 | G |
| 112018 | unique glycine-rich secreted protein | Hypothetical protein; | 2.462 | 0.113 | NA | -1.642 | D |
| 112258 | hypothetical protein | Hypothetical protein; | -0.118 | 3.117 | 0.106 | 3.486 | H |
| 119552 | unique secreted protein, S,P,R-rich | Hypothetical protein; | 0.36 | 3.995 | 0.304 | 4.364 | H |
| 119735 | glyceraldehyde-3-phosphate dehydrogenase | Metabolism; C-compound and carbohydrate metabolism; | -2.956 | -0.609 | -1.473 | 0.331 | F |
| 119759 | metR bZIP transcription factor | Transcription; Protein activity regulation; | -2.189 | 0.333 | -1.799 | 0.595 | F |
| 119819 | G-protein coupled receptor, mPR-type | Cellular communication, signal transduction mechanism; | -2.039 | -0.969 | -1.306 | 0.157 | F |
| 120017 | small oligopeptide transporter | Cellular transport, transport facilitation and transport routes; | 3.55 | -0.945 | 5.417 | 0.67 | E |
| 120031 | hypothetical protein | Hypothetical protein; | 3.859 | -0.197 | 3.474 | -0.938 | E |
| 120120 | GCN5-related N-acetyltransferase | Transcription; Protein activity regulation; | 0.538 | -2.966 | 1.75 | -0.243 | G |
| 120156 | cell morphogenesis protein PAG1 | Cell fate; | 2.436 | 0.143 | 2.134 | -0.452 | E |
| 120357 | putative Zn-binding oxidoreductase | Energy; | NA | 0.718 | 2.999 | 4.246 | C |
| 120568 | enolase | Metabolism; C-compound and carbohydrate metabolism; | -2.101 | -0.627 | -1.118 | 0.317 | F |
| 120784 | putative cell wall mannoprotein | Biogenesis of cellular components; | 2.393 | 1.108 | 0.123 | -1.628 | D |
| 120823 | secreted aldose epimerase | Metabolism; C-compound and carbohydrate metabolism; Biogenesis of cellular components; | 4.271 | -1.249 | 4.227 | -1.28 | E |
| 120873 | GH71 α-1,3-glucanase | Metabolism; C-compound and carbohydrate metabolism; | 3.108 | 0.868 | -0.173 | -2.514 | D |
| 120877 | protein of Zn-dependant ß-lactamase family | Hypothetical protein; | -0.065 | 1.215 | 1.32 | 3.48 | X |
| 120975 | hypothetical protein | Hypothetical protein; | 2.055 | NA | NA | -2.058 | D |
| 121136 | unique protein | Unique protein; | 0.497 | -0.319 | 3.223 | 3.563 | C |
| 121226 | 2-dehydropantoate-2-reductase | Metabolism; Metabolism of vitamins, cofactors, and prosthetic groups; | 1.291 | -2.386 | 2.537 | -0.7 | G |
| 121230 | unique protein with similarity to phosphoproteoglycans | Unique protein; | -2.389 | -2.454 | -0.027 | 0.139 | B |
| 121251 | putative cell wall protein (Metarhizium adhesion Mad1) | Biogenesis of cellular components; | -0.802 | -2.327 | 0.258 | -1.256 | G |
| 121308 | δ-1-pyrroline-5-carboxylate dehydrogenase | Energy; | 2.28 | 0.971 | 2.242 | 1.206 | E |
| 121412 | amino acid polyamine transporter II | Cellular transport, transport facilitation and transport routes; | 2.204 | 0.724 | 1.599 | -0.021 | E |
| 121415 | Zn2Cys6 transcription factor | Transcription; Protein activity regulation; | 2.211 | 2.185 | -0.043 | -0.131 | A |
| 121475 | unique protein | Unique protein; | 2.741 | 1.029 | 0.202 | -1.696 | D |
| 121486 | hypothetical protein | Hypothetical protein; | -3.612 | 1.325 | -3.219 | 1.463 | F |
| 121491 | trehalose synthase CCG9 | Metabolism; C-compound and carbohydrate metabolism; | -2.797 | -1.955 | -0.912 | 0.135 | B |
| 121534 | pyruvate decarboxylase | Metabolism; Amino acid metabolism; C-compound and carbohydrate metabolism; Energy | -5.326 | -2.263 | -2.396 | 0.775 | X |
| 121620 | NMT1 thiamine biosynthesis protein | Metabolism; Metabolism of vitamins, cofactors, and prosthetic groups; | -6.66 | -5.859 | -1.016 | -0.081 | B |
| 121735 | GH3 ß-glucosidase Cel3B | Metabolism; C-compound and carbohydrate metabolism; | 3.569 | 0.856 | 2.594 | -0.484 | E |
| 121746 | GH55 exo-ß-1,3-glucosidase ("GLUC78") | Metabolism; C-compound and carbohydrate metabolism; | 3.385 | 1.183 | 2.458 | 0.019 | E |
| 121818 | hypothetical secreted protein | Hypothetical protein; | -0.501 | -2.471 | 0.724 | -1.144 | G |
| 122127 | cell wall protein PhiA | Biogenesis of cellular components; Subcellular localization; | 1.909 | -1.245 | 4.523 | 1.447 | E |
| 122147 | unique protein | Unique protein; | 1.724 | -0.067 | -0.244 | -2.468 | D |
| 122242 | hypothetical protein | Hypothetical protein; | NA | -2.038 | 1.093 | 0.205 | G |
| 122271 | Zn2Cys6 transcription factor, high similarity to *Candida albicans* Fcr1 | Transcription; Protein with binding function or cofactor requirement; | 2.346 | 2.897 | NA | 0.771 | A |
| 122523 | hypothetical protein | Hypothetical protein; | -2.307 | -0.649 | -1.766 | -0.178 | F |
| 122780 | GH28 polygalacturonase | Metabolism; C-compound and carbohydrate metabolism; | 4.146 | -0.185 | 3.332 | -1.413 | E |
| 122811 | glutamine synthetase | Metabolism; Amino acid metabolism; | -2.431 | -0.382 | -0.08 | 2.095 | X |
| 123009 | glutamine synthetase | Metabolism; Amino acid metabolism; | 1.967 | -1.205 | 2.273 | -0.459 | E |
| 123079 | short chain dehydrogenase/reductase | Metabolism; Metabolism general; | 3.186 | 1.764 | NA | -0.384 | A |
| 123084 | (chloro)peroxidase | Metabolism; Metabolism general; | -6.911 | 0.073 | -6.581 | -0.031 | F |
| 123207 | hypothetical protein | Hypothetical protein; | 3.545 | -0.59 | 3.116 | -1.545 | E |
| 123234 | Coproporphyrinogen III oxidase | Protein fate; | 3.023 | 3.22 | NA | 0.229 | A |
| 123382 | subtilisin-type serine protease | Protein fate; | -2.708 | -1.506 | -0.962 | 0.057 | X |
| 123456 | GH65 αα'-trehalase | Metabolism; C-compound and carbohydrate metabolism; | 0.971 | -0.697 | 2.215 | 0.93 | E |
| 123468 | IMP dehydrogenase | Metabolism; Nucleotide metabolism; Cellular transport, transport facilitation and transport routes; | -2.738 | -0.12 | -2.446 | -0.347 | F |
| 123475 | Putative cell wall T-rich mannoprotein. Distantly related to *S. cerevisiae* Dan4p. | Biogenesis of cellular components; Subcellular localization; | -1.727 | -3.33 | 1.298 | -0.268 | B |
| 123713 | MedA | Transcription; Protein activity regulation; | 2.4 | -0.248 | 2.755 | 0.081 | E |
| 123718 | neutral amino acid permease | Metabolism; Amino acid metabolism; Cellular transport, transport facilitation and transport routes; | 0.482 | -2.246 | 5.007 | 2.457 | C |
| 123726 | amino acid transporter | Metabolism; Amino acid metabolism; Cellular transport, transport facilitation and transport routes; | 3.913 | 2.411 | 1.014 | -0.622 | A |
| 123786 | GH16 ß-1,3, ß-1,4-glucanase | Metabolism; C-compound and carbohydrate metabolism; | 3.423 | 0.679 | 0.95 | -2.536 | D |
| 123795 | allantoinase | Metabolism; Nucleotide metabolism; | -2.409 | -1.265 | 0.099 | 1.746 | C |
| 123914 | unique secreted protein | Unique protein; | -3.08 | NA | -3.448 | -1.207 | F |
| 123968 | hypothetical protein | Hypothetical protein; | 2.423 | 0.926 | 1.6 | -0.455 | E |
| 123976 | unique secreted protein | Unique protein; | 0.035 | -2.155 | 2.135 | 1.116 | C |
| 123978 | hypothetical methanol oxidase | Metabolism; Metabolism general; Protein with binding function or cofactor requirement; | 2.703 | 1.737 | -0.111 | -1.582 | D |
| 123979 | peptide transporter MTD1 | Cellular transport, transport facilitation and transport routes; | NA | 3.531 | NA | 4.499 | H |
| 124002 | MED2 nuclear component of the mediator complex | Transcription; Protein activity regulation; | NA | 1.57 | NA | 2.831 | H |
| 124043 | laccase, secreted | Metabolism; C-compound and carbohydrate metabolism; | 1.973 | NA | NA | -2.582 | D |
| 124079 | hypothetical protein | Hypothetical protein; | NA | 3.561 | NA | 4.867 | H |
| 124141 | hypothetical secreted protein with similarity to phosphoproteoglycan | Hypothetical protein; | 2.663 | -0.102 | 2.243 | -0.602 | E |
| 124277 | putative copper radical oxidase Cro1 | Protein with binding function or cofactor requirement; Cellular transport, transport facilitation and transport routes; | NA | 3.756 | NA | 4.3 | H |
| 124282 | hypothetical SET and MYND domain protein | Hypothetical protein; | -1.3 | -3.175 | 0.782 | -0.917 | G |
| 120117 | CRE1 | Transcription | -0.394 | 0.148 | -2.362 | -2.666 |  |

**Table S3:** Quantitative expression patterns determined by qRT-PCR of selected genes. Expression and p-values relate to the qPCR results

| **Protein ID** | **encoded protein** | **D [h-1]** | **Expression** | **p value** | **Arrays** | **qPCR** |
| --- | --- | --- | --- | --- | --- | --- |
|  |  |  |  |  |  |  |
| 21758 | isocitrate lyase | 0.07 | 49.609 | 0.001 | UP | UP |
| 23415 | amino acid permease DIP5 | 0.07 | 1984.41 | 0.001 | UP | UP |
|  |  | 0.025 | 1.624 | 0 | UP | UP |
| 46794 | MSF peptide transporter | 0.07 | 26.118 | 0.001 | UP | UP |
|  |  | 0.025 | 1.967 | 0.001 | UP | UP |
| 48211 | unknown conserved protein, intracellular | 0.07 | 10.116 | 0.001 | UP | UP |
|  |  | 0.025 | 2.444 | 0 | UP | UP |
| 49970 | H+ nucleoside cotransporter | 0.07 | 17.227 | 0.001 | UP | UP |
|  |  | 0.025 | 3.26 | 0.001 | UP | UP |
| 57749 | MSF transporter | 0.07 | 0.379 | 0 | DOWN | DOWN |
|  |  | 0.025 | 0.035 | 0.001 | DOWN | DOWN |
| 62380 | MSF major facilitator | 0.07 | 0.14 | 0.001 | DOWN | DOWN |
| 73818 | catalase C | 0.07 | 322.247 | 0.001 | UP | UP |
| 76215 | sulfide:quinone oxidoreductase | 0.07 | 3.488 | 0.001 | UP | UP |
| 76852 | GH2 glycoside hydrolase | 0.025 | 0.002 | 0.001 | DOWN | DOWN |
| 77093 | acid sphingomyelin phosphodiesterase | 0.07 | 0.594 | 0 | DOWN | DOWN |
|  |  | 0.025 | 0.049 | 0.001 | DOWN | DOWN |
| 120877 | Protein of Zn-ion ß-lactamase family | 0.07 | 3.59 | 0.001 | UP | UP |
|  |  | 0.025 | 12.907 | 0.002 | UP | UP |
| 122127 | cell wall protein PhiA | 0.07 | 90.788 | 0.001 | UP | UP |
|  |  | 0.025 | 2.299 | 0.001 | UP | UP |
| 124002 | MED2 component of the mediator complex | 0.07 | 53.011 | 0 | UP | UP |

**Table S4:** Enrichment analysis on FunCat categories. For each of the FunCat categories describe on Figure 4 we calculate for each gene cluster the enrichment ratio compared to the whole FunCat annotation. In addition, using the hypergeometric distribution we compute the statistical p-value associated to this enrichment.

|  | FunCat Categories | Biogenensis Of Cell Components | Cell Rescue, Defense And Virulence | Cellular Signalling | Transported Compounds | Protein Fate (Folding, Modification, Destination) | Transcription | Energy | Secondary Metabolites | Lipid, Fatty Acid And Isoprenoid Metabolism | C-Compound And Carbohydrate Metabolism | Aa Metabolism | General Metabolism |
| --- | --- | --- | --- | --- | --- | --- | --- | --- | --- | --- | --- | --- | --- |
| **Cluster** | **FunCat ID** | **42** | **32** | **30** | **20.01** | **14** | **11** | **02** | **01.20** | **01.06** | **01.05** | **01.01** | **01** |
| E | Enrichment | 0.35 |  | 1.04 | 1.64 | 0 | 0.28 | 0.72 | 0.96 | 0.21 | 0.97 | 0.82 | 0.05 |
|  | p-value | 4.8e-02 |  | 2.3e-01 | 2.2e-02 |  | 1.5e-02 | 1.9e-01 | 2.3e-01 | 3.1e-02 | 1.6e-01 | 2.2e-01 | 3.5e-02 |
| G | Enrichment | 0.48 | 0.41 |  | 1.03 | 0.62 | 0.39 |  |  | 0.56 | 1.33 |  | 0.15 |
|  | p-value | 2.6e-01 | 2.0e-01 |  | 2.5e-01 | 2.1e-01 | 1.8e-01 |  |  | 3.0e-01 | 1.9e-01 |  | 3.1e-01 |
| C | Enrichment | |  |  | 3.32 |  |  | 0.7 |  |  |  | 2.41 | 0.16 |
|  | p-value |  |  |  | 2.5e-04 |  |  | 3.5e-01 |  |  |  | 9.2e-02 | 3.3e-01 |
| H | Enrichment | 0.36 |  |  | 1.55 | 0.47 | 0.29 | 0.49 | 0.65 | 0.42 | 0.25 | 0.56 | 0.44 |
|  | p-value | 1.6e-01 |  |  | 1.0e-01 | 1.1e-01 | 9.5e-02 | 2.6e-01 | 3.4e-01 | 2.2e-01 | 5.6e-02 | 3.0e-01 | 1.2e-01 |
| D | Enrichment | 0.28 |  | 1.64 | 0.8 |  |  | 0.38 |  | 0.32 | 2.3 | 0.43 | 0.25 |
|  | p-value | 8.8e-02 |  | 1.7e-01 | 1.9e-01 |  |  | 1.8e-01 |  | 1.3e-01 | 1.8e-03 | 2.3e-01 | 2.4e-01 |
| F | Enrichment | | 0.39 | 0.89 | 1.13 |  | 0.55 | 1.23 | 1.64 |  | 0.62 | 0.35 | 0.14 |
|  | p-value |  | 6.4e-02 | 2.8e-01 | 1.6e-01 |  | 1.1e-01 | 1.9e-01 | 1.3e-01 |  | 1.1e-01 | 1.6e-01 | 1.7e-01 |
| B | Enrichment | 0.45 | 1.54 |  |  |  |  | 1.23 | 2.46 | 2.11 | 0.62 |  |  |
|  | p-value | 2.4e-01 | 1.5e-01 |  |  |  |  | 2.8e-01 | 8.9e-02 | 7.9e-02 | 2.1e-01 |  |  |
| A | Enrichment | |  |  | 0.82 | 0.49 | 0.61 |  | 0.69 | 0.44 | 1.83 | 0.59 | 0.12 |
|  | p-value |  |  |  | 2.3e-01 | 1.3e-01 | 2.0e-01 |  | 3.5e-01 | 2.3e-01 | 4.5e-02 | 3.2e-01 | 2.4e-01 |
| X | Enrichment | | 0.39 | 0.89 | 0.65 | 0.29 |  | 2.45 | 0.82 | 1.06 | 0.62 | 1.41 |  |
|  | p-value |  | 1.8e-01 | 3.8e-01 | 2.2e-01 | 9.3e-02 |  | 5.4e-02 | 3.7e-01 | 2.9e-01 | 2.1e-01 | 2.6e-01 |  |

**Table S5:** Complete enrichment analysis with Gene Ontologies. We perform a whole enrichment analysis for all the 171 annotated genes (among 250) detected as highly regulated targets using the Gene Ontology annotation of *T. reesei* from the JGI web site. We calculate the significance of the enrichment ratio using the hypergeometric distribution and the p-value where adjusted for multiple test using the Benjamini-Hochberg FDR correction. This table only display the category where adjusted p-values are below 0.05. There are 4,977 genes annotated with at least one Gene Ontology term among the 9,129 genes from the *Trichoderma reesei* genome.

| **GO term ID** | **GO description** | **GO category** | **Cre1** | **Total** | **Enrichment** | **Adjusted p-value** |
| --- | --- | --- | --- | --- | --- | --- |
| 8235 | oxidoreductase activity | molecular function | 38 | 452 | 2.4 | 2.09e-05 |
| 3077 | carbohydrate metabolism | biological process | 18 | 142 | 3.7 | 9.94e-05 |
| 7826 | membrane | cellular component | 32 | 383 | 2.4 | 9.94e-05 |
| 3879 | transport | biological process | 30 | 350 | 2.5 | 9.97e-05 |
| 2450 | transporter activity | molecular function | 23 | 251 | 2.7 | 5.04e-04 |
| 3922 | oligopeptide transport | biological process | 3 | 3 | 29.1 | 1.96e-03 |
| 3710 | fatty acid biosynthesis | biological process | 6 | 24 | 7.3 | 4.66e-03 |
| 18613 | glucan endo-1,3-alpha-glucosidase activity | molecular function | 3 | 4 | 21.8 | 5.38e-03 |
| 2653 | ATP binding | molecular function | 6 | 557 | 0.3 | 5.38e-03 |
| 18835 | regulation of oxidoreductase activity | biological process | 7 | 41 | 5 | 9.57e-03 |
| 2757 | nucleus | cellular component | 7 | 567 | 0.4 | 9.57e-03 |
| 7827 | integral to membrane | cellular component | 26 | 387 | 2 | 9.57e-03 |
| 1055 | nucleic acid binding | molecular function | 2 | 306 | 0.2 | 2.33e-02 |
| 1842 | chitinase activity | molecular function | 4 | 15 | 7.8 | 2.33e-02 |
| 5452 | endochitinase activity | molecular function | 4 | 15 | 7.8 | 2.33e-02 |
| 5149 | ammonium transporter activity | molecular function | 2 | 2 | 29.1 | 2.33e-02 |
| 4804 | metabolism | biological process | 30 | 519 | 1.7 | 2.36e-02 |
| 1829 | hydrolase activity, hydrolyzing O-glycosyl compounds | molecular function | 9 | 83 | 3.2 | 2.42e-02 |
| 5260 | carbohydrate transport | biological process | 7 | 53 | 3.8 | 2.42e-02 |
| 8471 | transferase activity | molecular function | 6 | 39 | 4.5 | 2.42e-02 |
| 17027 | cofactor binding | molecular function | 5 | 27 | 5.4 | 2.43e-02 |
| 3218 | electron transport | biological process | 22 | 349 | 1.8 | 2.74e-02 |
| 1056 | DNA binding | molecular function | 4 | 370 | 0.3 | 3.02e-02 |
| 2494 | amino acid-polyamine transporter activity | molecular function | 6 | 44 | 4 | 3.36e-02 |
| 2553 | sugar porter activity | molecular function | 7 | 59 | 3.5 | 3.36e-02 |
| 3929 | amino acid transport | biological process | 6 | 44 | 4 | 3.36e-02 |

**Table S6:** Primers used for construction of the *T. reesei* *Δcre1* strain.

| **Primer** | **Sequence** | **Fragment size [bp]** | **T_A_ [°C]** | **t_E_ [s]** |
| --- | --- | --- | --- | --- |
| cre5´F | CTCAAAGCCACGCAAATAGC | 1510 | 55 | 120 |
| cre5’Rtail-hph | GAGTATGTATCGCCGAAGAGGTGCTTTACAAGAGAGCCCAGAGAAG |
| M1LHhph | AGCACCTCTTCGGCGATAC | 2343 | 56 | 150 |
| M2LHhph | TCCTACCATTCCTTCTCTCG |
| cre3’Ftail-hph | ACCCTCGAGAGAAGGAATGGTAGGAACGACTTTGGGCTATAGATGG | 1496 | 55 | 120 |
| cre3´R | GTGCGTACATGAAGAAGATGG |

**Table S7:** Primers for Real Time quantification of selected genes.

| **Gene** | **Forward Primer (5’ to 3’)** | **Reverse Primer (5’ to 3’)** |
| --- | --- | --- |
| *tef* (reference gene) | CCACATTGCCTGCAAGTTCGC | GTCGGTGAAAGCCTCAACGCAC |
| ID122127 | TGATGCGGACCAGAACTTG | ACCCAGACATCCCTCATTCC |
| ID73818 | ACTTGGTTCCCGGCGTTGAG | TGAGAGGCGCATTGACAGGG |
| ID46794 | AGGGCTTCTTCACCATCGAC | AAGAAGACGGGCAGGAAGAG |
| ID62380 | CTTTGCCGTCACCTTTATTG | AAGATCAGGAAGCAGCAGAAC |
| ID120877 | GTGGACTGGATTCTGGAGAC | AATGGCGATGGATGGACTG |
| ID48211 | ACGAGGTCAAGTTCATTACGG | TCCCGTTGTTATCAGTGCC |
| ID76852 | CATCCTCTCCGACTCTGTAC | CCAAACATGAAGGTGAACTC |
| ID77093 | CGCAGGTTCCAACTACATC | GCGAGGGACAAATATACGAC |
| ID57749 | CTGCCCTGAACACTTACTGC | AACACCAATGCCCTCAATG |
| ID21758 | CATGGGCGTTGACTTGATC | GGTTCGTAGAGCCAAGGATG |
| ID49970 | ACGAGTACAATGCCTTCAACG | GGATACCCAGAGAGCCAATG |
| ID23415 | GTATTCGGCATCCTGACCTG | CACGAAGAACAGCGCAATG |
| ID76215 | CGACATTGCTGTTGTTGATC | GCCGTCGTTGTAGAACTTG |
| ID124002 | TCTCGCCTGCTATGTTATC | GCCACCGACAAATACTTC |
